# 5,7,12,14‐Tetrafunctionalized 6,13‐Diazapentacenes

**DOI:** 10.1002/chem.201904516

**Published:** 2019-12-16

**Authors:** Gaozhan Xie, Miriam Hauschild, Hendrik Hoffmann, Lukas Ahrens, Frank Rominger, Michal Borkowski, Tomasz Marszalek, Jan Freudenberg, Milan Kivala, Uwe H. F. Bunz

**Affiliations:** ^1^ Organisch-Chemisches Institut Ruprecht-Karls-Universität Heidelberg Im Neuenheimer Feld 270 69120 Heidelberg Germany; ^2^ Department of Molecular Physics Lodz University of Technology Zeromskiego 116 90924 Lodz Poland; ^3^ Max Planck Institute for Polymer Research Ackermannweg 10 55128 Mainz Germany; ^4^ Centre for Advanced Materials Im Neuenheimer Feld 225 69120 Heidelberg Germany

**Keywords:** diazapentacenes, heteroacenes, tetrasubstitution, X-ray diffraction

## Abstract

The synthesis, property evaluation, and single crystal X‐ray structures of four 5,7,12,14‐tetrafunctionalized diazapentacenes are presented. The synthesis of these compounds either starts from tetrabromo‐*N*,*N*‐dihydrodiazapentacene or from a diazapentacene tetraketone. Pd‐catalyzed coupling or addition of a lithium acetylide gave the precursors that furnish, after further redox reactions, the diazapentacenes as stable crystalline materials. The performance of the tetraphenyl‐substituted compound as n‐channel semiconductor was evaluated in organic field effect transistors.

## Graphical abstract

**Four in one strike**: The simple construction of fourfold substituted 6,13‐diazapentacenes by using Suzuki cross‐coupling or nucleophilic addition to a double quinone is described (see scheme).

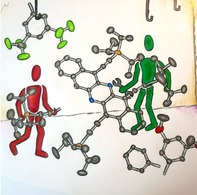

Herein, we describe the synthesis of tetrasubstituted 6,13‐diazapentacenes by using two different precursors. Azaacenes^1^ have aroused great interest, starting with the synthesis of the superb n‐channel semiconductor TIPS‐TAP.^2^, ^3^, ^4^ This interest was further stoked by new syntheses to construct azapentacenes^5^ to azaheptacenes,^6^ by using Pd‐catalyzed formation of embedded *N*,*N′*‐dihydropyrazines,^6^, ^7^ and the availability of several privileged, bis(tri‐*iso‐*propylsilylethynyl)‐substituted aromatic *ortho*‐diamines.^5^ These approaches lead to disubstituted azaacenes. The synthesis of higher substituted azaacenes (tetrasubstituted, hexasubstituted, etc.) is not common, although for their hydrocarbon analogues,^8^, ^9^, ^10^ some derivatives have recently been explored, including per‐substituted species furnishing twistacenes.^11^ Herein, we decorate the diazapentacene framework either by fourfold Suzuki–Miyaura coupling^12^ or by fourfold addition of a lithium acetylide. Reaction of the literature known tetrabromide **1** 13 with different boronic acids under standard palladium catalysis conditions gave the crude *N*,*N′*‐dihydro‐intermediates **2 a**–**c**, which were not further characterized but immediately oxidized by MnO_2_ into the target compounds **3 a**–**c** (53–79 % overall yield). The dihydro‐species **1** is much more soluble (and does not re‐oxidize the intermediately formed Pd^0^ species) than its oxidized heteroacene counterpart and was employed in our coupling reactions. Because **1** did not undergo Sonogashira reaction directly (see Scheme S1 in the Supporting Information for conditions), we obtained tetrayne **3 d** by reacting tetraone **4** with an excess of the lithium salt of TIPS acetylene and treatment of the intermediate with tin dichloride.^14^ Compound **5** was isolated in 26 % yield. Oxidation with MnO_2_ in acetonitrile then gave **3 d** in 95 % yield. Note that the electron‐withdrawing pyrazine units enable fourfold nucleophilic addition—TIPS acetylide only adds twice to the corresponding hydrocarbon tetraketone analogue.^15^, ^16^

Figure 1 displays the normalized absorption spectra of non‐fluorescent **3 a**–**d** (see also Table 1). We note that **3 a**–**c** display almost identical UV/Vis spectra despite the significant electronic differences in the substituents of **3 a**–**c**. The substituents only exert an inductive effect but do not increase the conjugation—not unexpected, because the arene groups are heavily twisted with respect to the diazapentacene backbone. Compound **3 d** with the four alkyne groups displays a 70–80 nm redshifted absorption at 743 nm, a consequence of the strong conjugation of the four alkyne groups with the diazapentacene nucleus (Scheme 1).^17^

**Figure 1:**
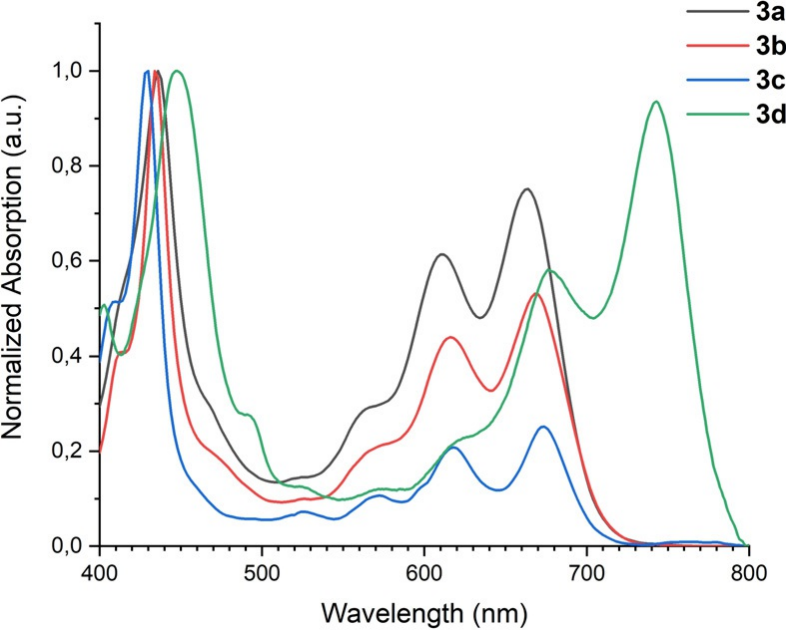
Normalized absorption spectra of **3 a**–**d** recorded in dichloromethane (DCM).

**Table 1:** Photophysical and electrochemical properties of **3 a**–**d**.

Compound	Abs_max_^[a]^ [nm]	*E*_ox1_^[b]^ [V]	*E*_red1_^[b]^ [V]	Ionization potential [eV] meas.^[c]^/calcd^[d]^	Electron affinity [eV] meas.^[c]^/calcd^[d]^	Gap [eV] meas.^[e]^/calcd^[e]^/opt.^[f]^
**3 a**	664	0.64	−1.33	−5.44/−5.08	−3.47/−2.98	1.97/2.10/1.69
**3 b**	668	0.63	−1.32	−5.43/−5.33	−3.48/−3.25	1.95/2.08/1.68
**3 c**	674	1.13	−1.14	−5.93/−6.27	−3.66/−4.23	2.27/2.04/1.72
**3 d**	743	0.77	−0.96	−5.57/–	−3.84/–	1.73/–/1.59
5,7,12,14‐tetraphenylpentacene	621^9^	–	–	–/−4.80^9^	–/−2.59^9^	–/2.21^9^/1.88^9^

[a] Absorption peaks in DCM. [b] First oxidation and reduction potentials measured in CV using ferrocene/ferrocenium as the reference redox system and internal standard (−4.8 eV vs. vacuum);^18^ [c] Calculated from CV measurements (*E*_HOMO_=−4.80 eV−*E*_ox1_; *E*_LUMO_=−4.80 eV−*E*_red1_). [d] Calculated with Gaussian 09 B3LYP/6‐311++G**//DFT/B3LYP/6‐31+G**.^19^ [e] Estimated from *E*_HOMO_ and *E*_LUMO_ (*E*_gap_=*E*_LUMO_–*E*_HOMO_). [f] Estimated from absorption onset recorded in DCM.

**Scheme 1:**
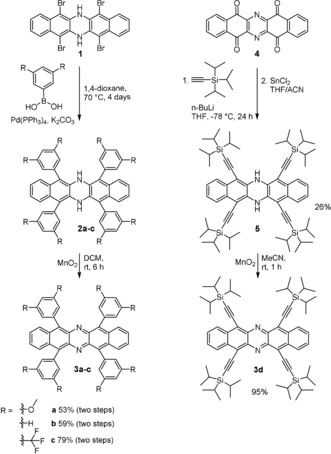
Synthesis of substituted diazapentacenes **3 a**–**d**.

Compounds **3 a**–**d** were investigated by cyclic voltammetry (Table 1). They can be both oxidized and reduced, suggesting ambipolar behavior.^20^ As was expected, **3 d** and **c** display the highest oxidation potential. The effect is particularly strong for **3 c**, featuring four CF_3_ groups. The same trend was observed for the reduction potentials, which are −1.14 V for **3 c** and −0.96 V for **3 d**. The electron affinity for **3 c** and **d** are estimated to be −3.7 and −3.8 eV, respectively. Although the alkyne substituents influence HOMO and LUMO position differently and lead to a decreased electrochemical and optical gap, electron withdrawing substituents on the aryl groups in **3 c** stabilize both frontier molecular orbitals (FMOs) similarly. In comparison to 5,7,12,14‐tetraphenylpentacene,^9^ nitrogen substitution leads to decreased FMO energy levels, as was expected.

Compounds **3 a**–**d** form suitable specimens useful for X‐ray single crystal analysis (Figure 2 and the Supporting Information). In compounds **3 a**–**c**, the diazapentacene backbone is planar, and the four aryl groups are oriented parallel to each other and considerably twisted with respect to the diazapentacene (dihedral angles: 63° and 65° for **3 a**; 57° and 61° for **3 b** and 66° and 71° for **3 c**). The molecules of **3 a** and **c** pack in a herringbone pattern with no π–π overlap between the molecules. The molecules of **3 b** pack in π–π stacked dimers with an interplanar distance of 3.60 Å, which are arranged in one‐dimensional slipped stacks. In the case of **3 d**, the four TIPS‐ethynyl groups crowd each other. This leads to a twist of the diazapentacene nucleus with an end‐to‐end torsion angle of 20°.

**Figure 2:**
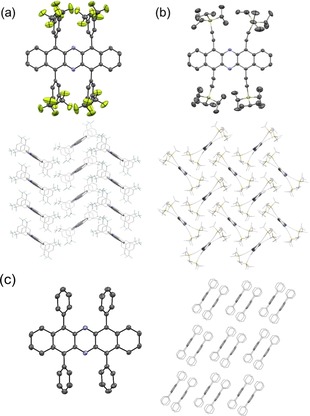
Molecular structures and solid‐state packings of (a) **3 c**, (b) **3 d**, and (c) **3 b** (hydrogen atoms omitted for clarity).

The steric crowding of the four TIPS groups also enforces a bend in the alkynes away from each other, even though direct *peri* interactions are not present due to the pyrazine unit interspersed between the alkyne‐carrying rings. Compound **3 d** also packs in a herringbone motif; here also, as was expected,^21^ π–π overlap is absent. The observed packing suggests that larger acenes, for example, diazaheptacenes,^22^ might be stabilized in the solid state with the current substituent pattern and at the same time display attractive solid‐state ordering that would allow their use in ambipolar transistors.

Next issue to address was stability of the diazapentacenes compared to their hydrocarbon analogues. The stability of 5,7,12,14‐tetraphenylpentacene was assessed through UV/Vis measurements in dilute solution—it photooxidized in toluene^10^ or dichloromethane^9^ under ambient conditions (light and air) in less than 20 minutes via *endo*‐peroxide formation. Nitrogen substitution protected the system. The absorption profile of alkynylated **3 d** remains unchanged for 24 hours, photooxidation of **3 a**–**c** depends on the electronic demand of the aryl substituents. Electron‐deficient trifluoromethyl groups stabilize the system most (14 % absorption loss after 24 h), but even electron‐rich, dimethoxy‐substituted **3 a** was still fairly stable (50 % loss after 24 h).

To initially evaluate the potential of the newly synthesized tetrasubstituted diazapentacenes as n‐channel organic semiconductors, organic field‐effect transistors (OFETs) were fabricated by physical vapor deposition of **3 b** (for details regarding the device fabrication, see the Supporting Information). The compound showed n‐type charge transport behavior with a maximum electron mobility of 3.2×10^−3^ cm^2^ V^−1^ s^−1^, threshold voltage 30 V and on/off ratio on the level of 10^4^. The average charge carrier mobility calculated for twelve transistors was 1.76×10^−3^±0.51 cm^2^ V^−1^ s^−1^. In contrast, for parent unsubstituted 6,13‐diazapentacene hole mobilities in a range of 10^−5^ were reported.^3^ This finding clearly highlights the beneficial impact of the 5,7,12,14‐substitution pattern on the n‐channel device performance and constitutes an asset for our future efforts in this area.

In conclusion, we developed symmetrically tetrafunctionalized 6,13‐diazapentacenes, either starting from bisquinone **4** or from the *N*,*N′*‐dihydro‐tetrabromide **1**. Both routes work well and give the expected products in reasonable yields. The compounds are stabilized with respect to photooxidation and the tetraphenyl‐substituted representative **3 b** shows n‐channel behavior. In future, we will expand this concept to 6,7,14,15‐tetraazahexacene and to 7,16‐diazaheptacene. Herein, the solubility of the precursors might be a problem, but the prospect of stable diazaheptacenes is particularly attractive.^23^

## Experimental Section

6,13‐Dihydro‐6,13‐diazapentacene and 5,7,12,14‐tetrabromo‐6,13‐dihydro‐6,13‐diazapentacene were synthesized by literature procedure.^13^, ^24^

### General synthesis procedure for 3 a–c

5,7,12,14‐Tetrabromo‐6,13‐dihydro‐6,13‐diazapentacene **1** (200 mg, 0.33 mmol), arylboronic acid (8.00 equiv), Pd(PPh_3_)_4_ (76.0 mg, 65.8 μmol, 0.20 equiv), and K_2_CO_3_ (462 mg, 3.34 mmol, 10.0 equiv) were added into the flask under N_2_. 1,4‐Dioxane (16 mL) and water (4 mL) were purged with N_2_ for 20 min and then added into the flask. The resulting mixture was stirred at 70 °C for 4 days. After cooling to room temperature (rt), a pale green precipitate was formed. It was collected by filtration and washed with water and ethanol. The crude product was dissolved in DCM (20 mL), followed by treatment with MnO_2_ (872 mg, 10.0 mmol, 30.0 equiv.) at rt for 6 h.

**5,7,12,14‐Tetra(3,5‐dimethoxylphenyl)‐6,13‐diazapentacene (3 a)**: 3,5‐Dimethoxylphenylboronic acid (487 mg, 2.56 mmol, 8.00 equiv) was employed. After reaction, DCM was evaporated under reduced pressure and the crude product was purified by flash column chromatography (SiO_2_, ethyl acetate (EE)) to give a dark green solid. Further washing with petroleum ether (PE) gave pure **3 a**. Yield: 146 mg, 0.18 mmol, 53 %. Melting point (M.p.): >400 °C (decomp). ^1^H NMR (CDCl_3_, 500 MHz, 295 K): *δ*=8.15–7.98 (m, 4 H), 7.42–7.32 (m, 4 H), 6.67–6.47 (m, 12 H), 3.78–3.66 (m, 24 H) ppm. ^13^C {^1^H} NMR (CDCl_3_, 126 MHz, 295 K): *δ*=160.2, 138.1, 133.2, 127.8, 127.2, 110.2, 100.2, 55.4 ppm. IR: $\tilde{\nu }$ =3073, 2993, 2932, 2829, 1594, 1449, 1206, 1137, 1057, 757 cm^−1^. MS (MALDI) *m*/*z*: [*M*]^+^: calcd for C_52_H_44_N_2_O_8_: 824.9300; found 825.3180; correct isotope distribution.

**5,7,12,14‐Tetraphenyl‐6,13‐diazapentacene (3 b)**: Phenylboronic acid (326 mg, 2.56 mmol, 8.00 equiv) was employed. After reaction, DCM was evaporated under reduced pressure, and the crude product was purified by flash column chromatography (SiO_2_, DCM) to give a dark green solid. Subsequent washing with EE gave pure **3 b**. Yield: 116 mg, 0.20 mmol, 59 %. M.p.: >400 °C (decomp). ^1^H NMR (CD_2_Cl_2_, 600 MHz, 295 K): *δ*=8.00–7.94 (m, 4 H), 7.50–7.46 (m, 8 H), 7.45–7.40 (m, 12 H), 7.33–7.29 (m, 4 H) ppm. ^13^C {^1^H} NMR (CD_2_Cl_2_, 151 MHz, 295 K): *δ*=138.9, 138.8, 137.4, 133.2, 132.8, 128.1, 128.0, 127.5, 126.7 ppm. IR: $\tilde{\nu }$ =3080, 3053, 3023, 1730, 1434, 1392, 761, 697, 476 cm^−1^. MS (ESI) *m*/*z*: [*M*+H]^+^: calcd for C_44_H_29_N_2_^+^: 585.7300; found 585.2330; *m*/*z*: [*M*+Na]^+^: calcd for C_44_H_28_N_2_Na^+^: 607.7118; found 607.2149; correct isotope distribution.

**5,7,12,14‐Tetra(3,5‐bis(trifluoromethyl)phenyl)‐6,13‐diazapentacene (3 c)**: 3,5‐Bis(trifluoromethyl)phenylboronic acid (690 mg, 2.56 mmol, 8.00 equiv) was employed. After reaction, DCM was evaporated under reduced pressure, and the crude product was purified by a flash column chromatography (SiO_2_, PE/DCM 3:1) to give a dark green solid. After washing by petroleum ether (PE), pure **3 c** was obtained. Yield: 298 mg, 0.26 mmol, 79 %. M.p.: >400 °C (decomp). ^1^H NMR (CD_2_Cl_2_, 600 MHz, 295 K): *δ*=8.00–7.97 (m, 4 H), 7.86–7.84 (m, 8 H), 7.80–7.76 (m, 4 H), 7.49–7.45 (m, 4 H) ppm. ^13^C {^1^H} NMR (CD_2_Cl_2_, 126 MHz, 295 K): *δ*=139.0, 138.7, 136.5, 133.7, 132.5, 131.6, 128.7, 127.0, 124.8, 123.0, 122.2, 121.2 ppm. IR: $\tilde{\nu }$ =2920, 2844, 1609, 1502, 1263, 1008, 974, 826, 552 cm^−1^. MS (MALDI) *m*/*z*: [*M*]^+^: calcd for C_52_H_20_N_2_F_24_: 1128.7007; found 1128.1257; correct isotope distribution.

**5,7,12,14‐Tetrakis(triisopropylsilylacatylene)‐6,13‐dihydro‐6,13‐diazapentacene (5)**: TIPS acetylene (3.63 mL, 16.2 mmol, 11.0 equiv) was dissolved in dry THF (30 mL). Subsequently, *n*BuLi (2.5 m in hexanes, 5.88 mL, 14.7 mmol, 10 equiv) of was added at −70 °C, and the mixture was stirred for 2 h at −70 °C. After this time, **4** (500 mg, 1.47 mmol, 1.00 equiv) was added, the reaction mixture was brought to room temperature, and stirred for 16 h. The solvent was removed under reduced pressure, and the precipitate was filtered through a SiO_2_ pad first with PE and afterwards with EE. The solvent of the EE fraction was removed under reduced pressure, and the resulting colorless solid was dissolved in acetonitrile (5 mL), and SnCl_2_⋅2 H_2_O (4.00 g) was added. The reaction mixture was stirred for 1 h at room temperature. The solvent was removed under reduced pressure and purification by column chromatography (SiO_2_, PE/DCM 4:1) gave 387 mg (386 μmol, 26 %) of **5** as a yellow solid. M.p.: >350 °C (decomp). ^1^H NMR (CD_2_Cl_2_, 600 MHz, 295 K): *δ*=7.98–7.96 (m, 4 H), 7.28 (s, 2 H), 7.28–7.26 (m, 4 H), 1.31–1.17 (m, 84 H) ppm. ^13^C {^1^H} NMR (CD_2_Cl_2_, 151 MHz, 295 K): *δ*=132.5, 130.8, 125.7, 125.3, 106.0, 100.6, 100.5, 19.1, 12.5 ppm. IR: $\tilde{\nu }$ =3370, 2940. 2862, 2124, 1590, 1526, 1468, 1425, 1407, 1381, 1349, 1254, 1154, 1070, 996, 917, 881, 753, 674, 658, 640, 526, 502 cm^−1^. MS (DART+) *m*/*z*: [*M*+H]^+^: calcd for C_64_H_95_N_2_Si_4_^+^: 1003.6567; found 1003.6514; correct isotope distribution.

**5,7,12,14‐Tetrakis(triisopropylsilylacatylene)‐6,13‐diazapentacene (3 d)**: Compound **5** (200 mg, 199 μmol, 1.00 equiv) was dissolved in DCM (20 mL), followed by treatment with MnO_2_ (520 mg, 5.98 mmol, 30.0 equiv) at room temperature for 6 h. The solvent was removed under reduced pressure, and purification by column chromatography (SiO_2_, PE/DCM 4:1) gave 190 mg (190 μmol, 95 %) of **3 d** as a dark green solid. M.p.: >350 °C (decomp). ^1^H NMR (CD_2_Cl_2_, 400 MHz, 295 K): *δ*=8.80–8.78 (m, 4 H), 7.64–7.61 (m, 4 H), 1.31–1.41 (m, 14 H), 1.23–1.31 (m, 71 H), 1.41–1.22 (m, 84 H) ppm. ^13^C {^1^H} NMR (CD_2_Cl_2_, 101 MHz, 295 K): *δ*=140.9, 137.5, 128.7, 128.4, 121.2, 110.5, 104.2, 19.2, 12.4 ppm. IR: $\tilde{\nu }$ =2940, 2888, 2862, 2123, 1525, 1460, 1439, 1428, 1391, 1366, 1234, 1121, 1101, 1074, 1052, 1015, 995, 919, 881, 856, 758, 672, 655, 641, 566, 513, 506, 482, 459, 452, 442, 410 cm^−1^. MS (DART^+^) *m*/*z*: [*M*+H]^+^: calcd for C_64_H_93_N_2_Si_4_^+^: 1001.6410; found 1001.6369; correct isotope distribution.

Crystallographic data: CCDC https://www.ccdc.cam.ac.uk/services/strctures?id=doi:10.1002/chem.201904516 contain the supplementary crystallographic data for this paper. These data are provided free of charge by http://www.ccdc.cam.ac.uk/

## Conflict of interest

The authors declare no conflict of interest.

## Supporting information

As a service to our authors and readers, this journal provides supporting information supplied by the authors. Such materials are peer reviewed and may be re‐organized for online delivery, but are not copy‐edited or typeset. Technical support issues arising from supporting information (other than missing files) should be addressed to the authors.

Supplementary
