# Supplementary material for: 5,7,12,14‐Tetrafunctionalized 6,13‐Diazapentacenes
Source: Chemistry. 2019 Dec 16;26(4):799–803. doi: 10.1002/chem.201904516 (PMC7004126; doi:10.1002/chem.201904516)
Supplement: Supplementary file 1 — Supplementary [file CHEM-26-799-s001.pdf]

CHEMISTRY
A European Journal

Supporting Information

5,7,12,14-Tetrafunctionalized 6,13-Diazapentacenes

Gaozhan Xie,[a] Miriam Hauschild,[a] Hendrik Hoffmann,[a] Lukas Ahrens,[a] Frank Rominger,[a] Michal Borkowski,[b] Tomasz Marszalek,[b, c] Jan Freudenberg,*[a] Milan Kivala,*[a, d] and Uwe H. F. Bunz*[a]

chem_201904516_sm_miscellaneous_information.pdf

Index:

S1. Instruments and Methods 02

S2. Syntheses 03

S3. Absorption Spectra 04

S4. Electrochemistry 05

S5. Computational Studies 06

S6. NMR Spectroscopy 07

S7. Mass Spectrometry 13

S8. Infrared Spectroscopy 16

S9. Crystal Structures 17

S10. Device Fabrication 22

S11. References 23

S12. Cartesian Coordinates of Computational Studies . 23

S1. Instruments and Methods

Thin-layer chromatography (TLC) was carried out on Polygram SILG/UV254 plates from Macherey, Nagel&Co.KG (Düren, Germany) and examined under ultraviolet irradiation (254 and 365 nm). NMR spectra (^{1}H, ^{13}C) were recorded at Bruker Avance III 300, Bruker Avance III 400 or Bruker Avance III 600. Chemical shifts (δ) are given in parts per million (ppm) relative to internal solvent signals. The following abbreviations describe the signal multiplicities: s = single m = multiplet. IR spectra were recorded on a JASCO FT/IR-4100. High resolution mass spectra (HR-MS) were obtained from electrospray ionization (ESI) or matrix-assisted laser desorption/ionization (MALDI) or direct analysis in real time (DART) experiments. UV-vis spectra were recorded on a Jasco V670. Crystal structure analysis was accomplished on Bruker Smart CCD or Bruker APEX diffractometers. CV measurements were performed on a VersaSTAT 3 potentiostat by Princeton Applied Research. Computational studies were carried out using DFT calculations on Gaussian 09. Geometry optimizations were performed using the B3LYP functional basis set. At this geometry, the absolute energy and FMO energies were assigned by a single-point approach at the B3LYP/6-311++G** level.[1]

S2. Syntheses

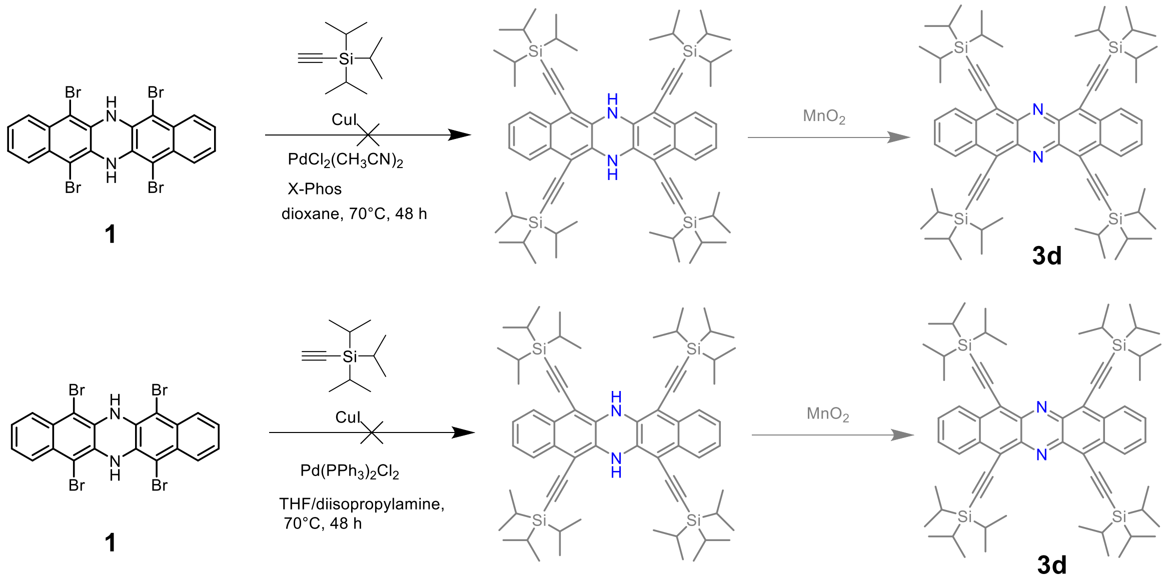

Scheme S1. Sonogashira coupling towards **3d** using tetrabromide **1** was not met with success.

6,13-Diazapentacene-5,7,12,14-tetraone (**4**)

A mixture of 6,13-dihydro-6,13-diazapentacene 1.00 g (3.54 mmol, 1.00 eq.), sodium dichromate (10.4 g, 35.4 mmol) and 120 mL acetic acid were refluxed for 60 min. After the reaction was cooled to room temperature, the precipitate was filtered, washed with water and dried. 738 mg (2.17 mmol, 61%) of **4** was obtained as a yellow solid. Mp: > 300 °C (dec.).. ^{1}H NMR (CD_3SOCD_3, 400 MHz, 295 K): δ = 8.34 (m, 4H), 8.04 (m, 4H), ppm. $^{13}C\{^{1}H\}$ NMR (CD_3SOCD_3, 101 MHz, 295 K): δ = 180.2, 146.8, 135.0, 133.6, 127.2, ppm. IR: $\tilde{\nu}$ = 3350, 3069, 1693, 1584, 1359, 1248, 1202, 1158, 1044, 997, 981, 801, 701, 668, 440 cm^{-1}. HRMS (DART$^+$) m/z: $[M+H]^+$: calcd. for $C_{40}H_{20}N_5O_8^+$: 698.1306; found 698.1286; correct isotope distribution.

S3. Absorption Spectra

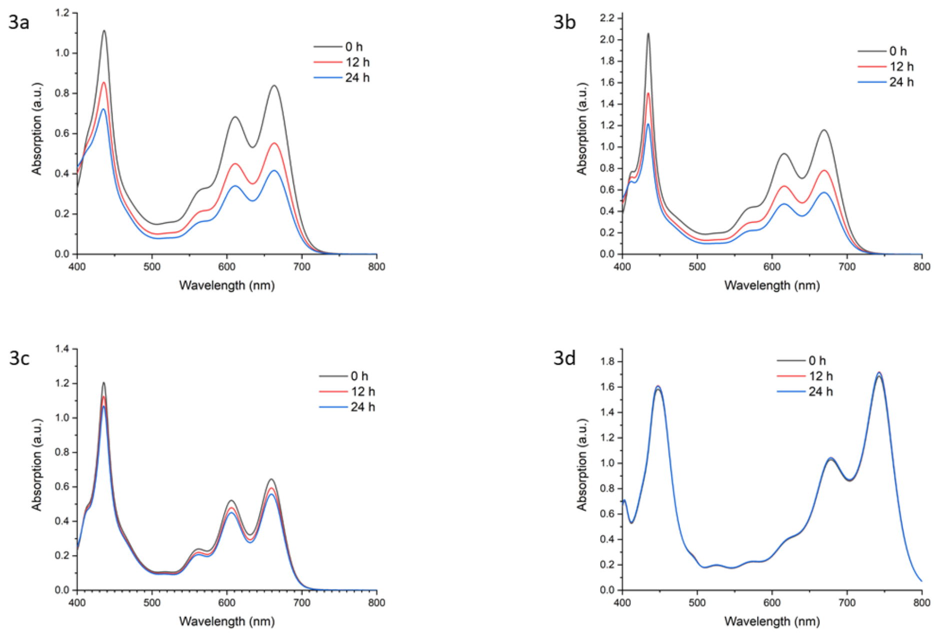

Figure S1. Stability measurements: **3a**, **3b**, **3c**, and **3d** measured in dichloromethane (DCM) c = 10^{-4} M at 25 °C exposed to ambient light and air after 12 h and 24 h.

S4. Electrochemistry

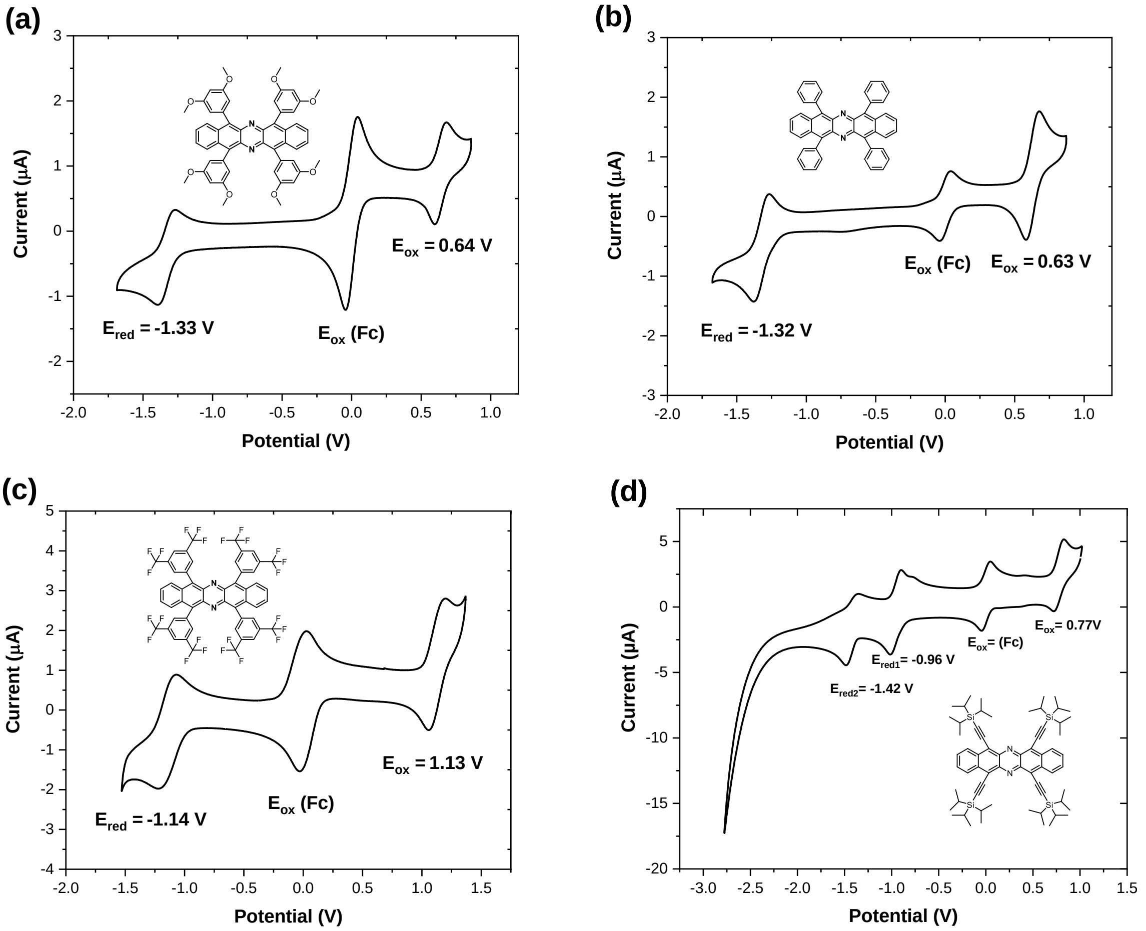

Figure S2. Cyclic voltammograms of a) **3a**, b) **3b**, c) **3c**, and d) **3d** using a gold working electrode, a platinum/titanium wire auxiliary electrode, and a silver wire reference electrode in degassed 0.1 M NBu_4PF_6-dichloromethane solution, and ferrocene/ferrocenium as the reference redox system and internal standard (−4.8 eV).[2]

S5. Computational Study

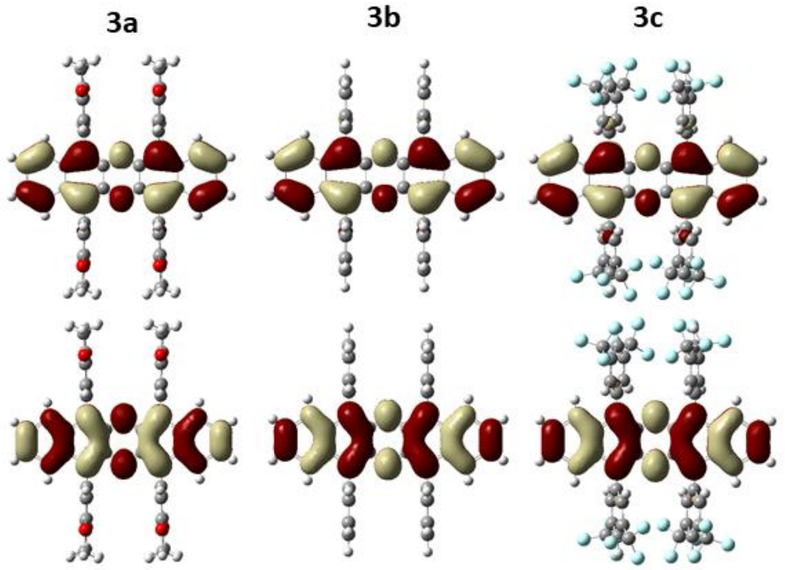

Figure S3. Frontier molecular orbitals calculated at DFT/B3LYP/6-311++G** level.

S6. NMR Spectroscopy

3a

^{1}H NMR

Acquisition Time (sec)	3.2768	***Comment***	Z810701_0032 (PA BBI 500S2 H-BB-D-05 Z)			***D***	0.1	***D1***	0.1
DE	6.5	***DS***	2	***Date***	29 Oct 2018 16:57:12			***Date Stamp***	29 Oct 2018 16:57:12
File Name	C:\Users\bunz\Application Data\SSH\temp\1\PDATA\1\1r					***Frequency (MHz)***	500.1301	***GB***	0
INSTRUM	<spect>	***LB***	0.3	***NS***	512	***Nucleus***	1H	***Number of Transients***	512
Origin	spect	***Original Points Count***	49152	***Owner***	ns	***PC***	1		
PROBHD	<Z810701_0032 (PA BBI 500S2 H-BB-D-05 Z)>			***PULPROG***	<zg30>	***Points Count***	65536	***Pulse Sequence***	zg30
Receiver Gain	322.00	***SF***	500.130050707952			***SFO1***	500.13250065		
SI	65536	***SSB***	0	***SW(cyclical) (Hz)***	15000.00	***SWH***	15000	***Solvent***	CHLOROFORM-d
Spectrum Offset (Hz)	2488.5029	***Spectrum Type***	standard	***Sweep Width (Hz)***	14999.77	***TD***	98304	***TD0***	64
TE	294.9941	***Temperature (degree C)***	21.994	***UNC1***	<1H>	***WDW***	1		

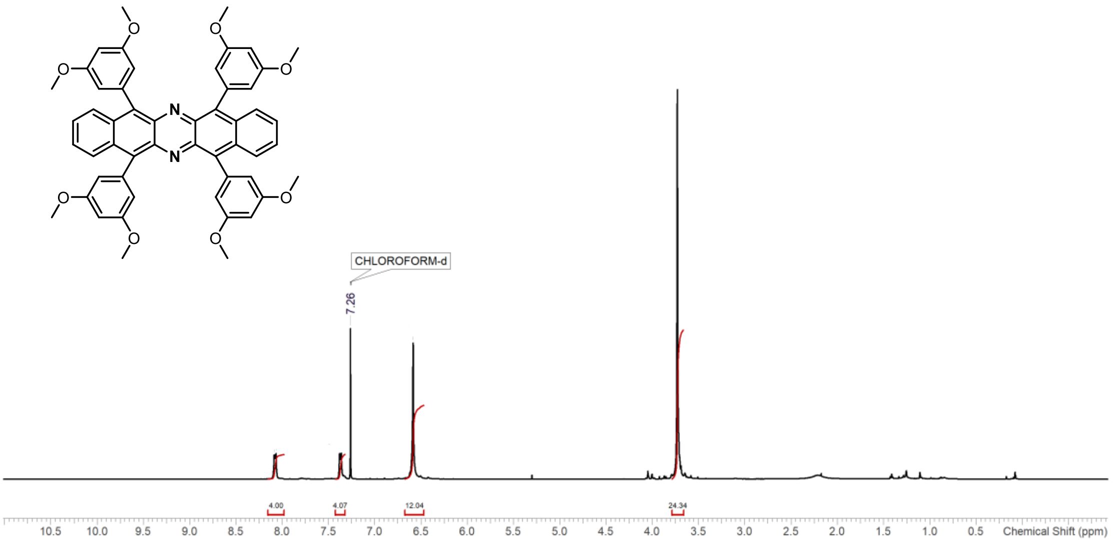

^{13}C{^{1}H} NMR

Acquisition Time (sec)	1.7302	***Comment***	Z810701_0032 (PA BBI 500S2 H-BB-D-05 Z)			***D***	1.5	***D1***	1.5
DE	20	***DS***	4	***Date***	29 Oct 2018 21:49:10			***Date Stamp***	29 Oct 2018 21:49:10
File Name	C:\Users\bunz\Application Data\SSH\temp\2\PDATA\1\1r					***Frequency (MHz)***	125.7578	***GB***	0
INSTRUM	<spect>	***LB***	0.5	***NS***	3200	***Nucleus***	13C	***Number of Transients***	3200
Origin	spect	***Original Points Count***	65536	***Owner***	ns	***PC***	1.4		
PROBHD	<Z810701_0032 (PA BBI 500S2 H-BB-D-05 Z)>			***PULPROG***	<zgpg30>	***Points Count***	65536	***Pulse Sequence***	zgpg30
Receiver Gain	2050.00	***SF***	125.757789	***SFO1***	125.77162235679			***SI***	65536
SSB	0	***SW(cyclical) (Hz)***	37878.79	***SWH***	37878.7878787879			***Solvent***	CHLOROFORM-d
Spectrum Offset (Hz)	13850.2148	***Spectrum Type***	standard	***Sweep Width (Hz)***	37878.21	***TD***	131072	***TD0***	400
TE	295.0027	***Temperature (degree C)***	22.003	***UNC1***	<13C>	***WDW***	1		

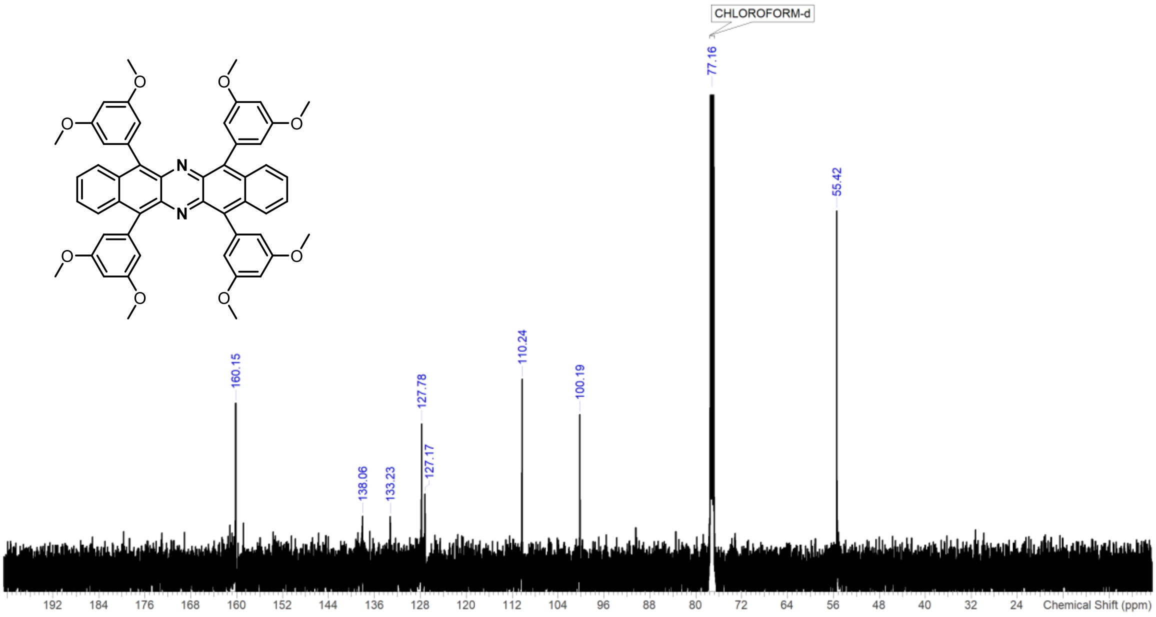

3b

^{1}H NMR

Multiplets Integrals Sum	28.29	Number of Nuclei	28 H's

**Acquisition Time (sec)**	3.6351	**Comment**	GZ 273	**D**	0.1	**D1**	0.1	**DE**	12
**DS**	2	**Date**	13 Apr 2019 21:57:39			**Date Stamp**	13 Apr 2019 21:57:39		
**File Name**	\\bunz22\Mitarbeiter\Gaozhan XIE\03_analytics\01-NMR\nmr02\e190412ubgz.273\2\PDATA\1\1r					**Frequency (MHz)**	600.2438	**GB**	0
**INSTRUM**	<spect>	**LB**	0.3	**NS**	128	**Nucleus**	1H	**Number of Transients**	128
**Origin**	spect	**Original Points Count**	65536	**Owner**	ns	**PC**	1		
**PROBHD**	<Z132808_0001 (CP QCI 600S3 H/P/C-N-D-05 Z LT)>					**PULPROG**	<zg30>	**Points Count**	65536
**Pulse Sequence**	zg30	**Receiver Gain**	16.84	**SF**	600.243829	**SFO1**	600.246830219145		
**SI**	65536	**SSB**	0	**SW(cyclical) (Hz)**	18028.85	**SWH**	18028.8461538462		
**Solvent**	DICHLOROMETHANE-d2			**Spectrum Offset (Hz)**	2807.2170	**Spectrum Type**	standard	**Sweep Width (Hz)**	18028.57
**TD**	131072	**TD0**	16	**TE**	295.0002	**Temperature (degree C)**	22.000	**UNC1**	<1H>
**WDW**	1								

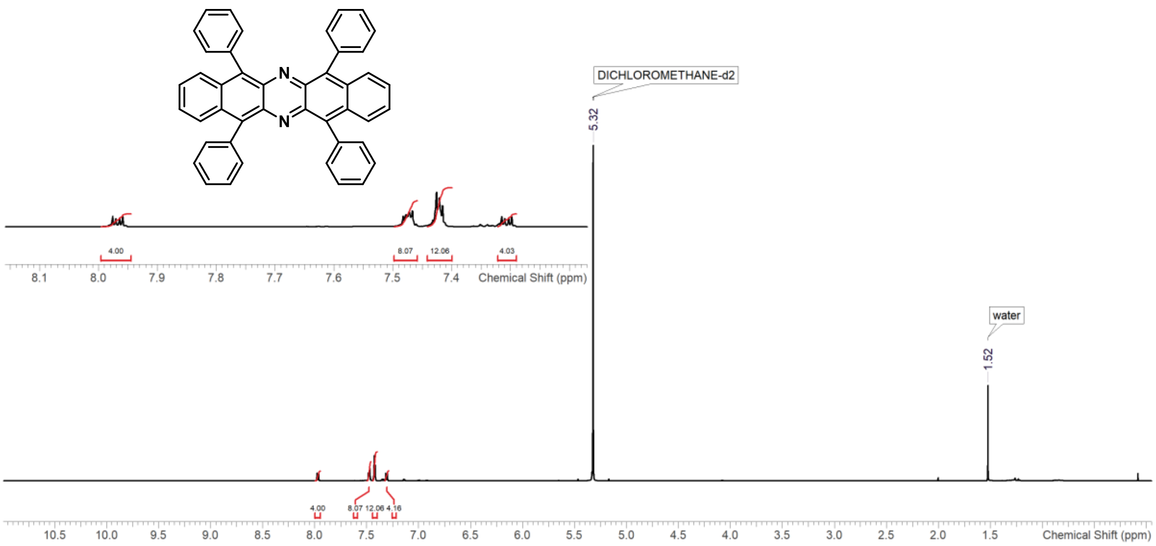

^{13}C{^{1}H} NMR

Multiplets Integrals Sum	0.00	Number of Nuclei	0 C's

**Acquisition Time (sec)**	1.0795	**Comment**	GZ 273	**D**	0.03	**D1**	2	**DE**	18
**DS**	4	**Date**	13 Apr 2019 21:48:39			**Date Stamp**	13 Apr 2019 21:48:39		
**File Name**	\\bunz22\Mitarbeiter\Gaozhan XIE\03_analytics\01-NMR\nmr02\e190412ubgz.273\1\PDATA\1\1r					**Frequency (MHz)**	150.9314	**GB**	0
**INSTRUM**	<spect>	**LB**	1	**NS**	3200	**Nucleus**	13C	**Number of Transients**	3200
**Origin**	spect	**Original Points Count**	49066	**Owner**	ns	**PC**	1.4		
**PROBHD**	<Z132808_0001 (CP QCI 600S3 H/P/C-N-D-05 Z LT)>			**PULPROG**	<zgpg30>	**Points Count**	65536	**Pulse Sequence**	zgpg30
**Receiver Gain**	2050.00	**SF**	150.931431	**SFO1**	150.94803345741			**SI**	65536
**SSB**	0	**SW(cyclical) (Hz)**	45454.55	**SWH**	45454.5454545455				
**Solvent**	DICHLOROMETHANE-d2			**Spectrum Offset (Hz)**	16644.2480	**Spectrum Type**	standard	**Sweep Width (Hz)**	45453.85
**TD**	98132	**TD0**	400	**TE**	295.0001	**Temperature (degree C)**	22.000	**UNC1**	<13C>
**WDW**	1								

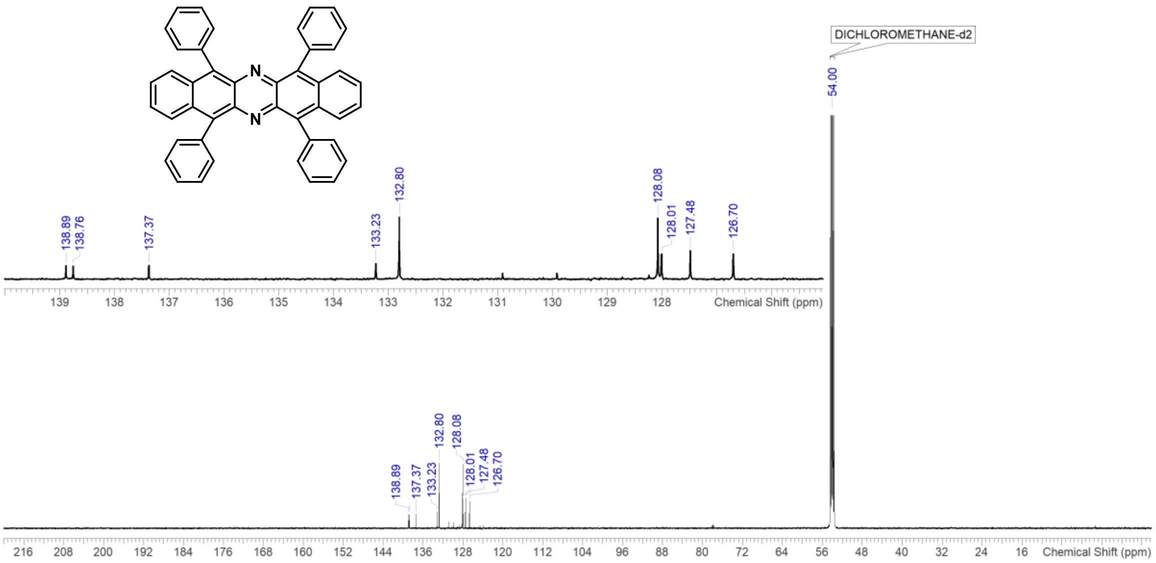

3c

^{1}H NMR

Multiplets Integrals Sum	19.81	Number of Nuclei	20 H's

**Acquisition Time (sec)**	3.6351	**Comment**	GZ 399	**D**	0.1	**D1**	0.1	**DE**	12
**DS**	2	**Date**	25 Apr 2019 02:14:00			**Date Stamp**	25 Apr 2019 02:14:00		
**File Name**	\\bunz22\Mitarbeiter\Gaozhan XIE\03_analytics\01-NMR\nmr02\e190424ubgz.399\2\PDATA\1\1r					**Frequency (MHz)**	600.2438	**GB**	0
**INSTRUM**	<spect>	**LB**	0.3	**NS**	128	**Nucleus**	1H	**Number of Transients**	128
**Origin**	spect	**Original Points Count**	65536	**Owner**	ns	**PC**	1		
**PROBHD**	<Z132808_0001 (CP QCI 600S3 H/P/C-N-D-05 Z LT)>					**PULPROG**	<zg30>	**Points Count**	65536
**Pulse Sequence**	zg30	**Receiver Gain**	16.84	**SF**	600.243829	**SFO1**	600.246830219145		
**SI**	65536	**SSB**	0	**SW(cyclical) (Hz)**	18028.85	**SWH**	18028.8461538462		
**Solvent**	DICHLOROMETHANE-d2			**Spectrum Offset (Hz)**	2807.2170	**Spectrum Type**	standard	**Sweep Width (Hz)**	18028.57
**TD**	131072	**TD0**	16	**TE**	294.9995	**Temperature (degree C)**	22.000	**UNC1**	<1H>
**WDW**	1								

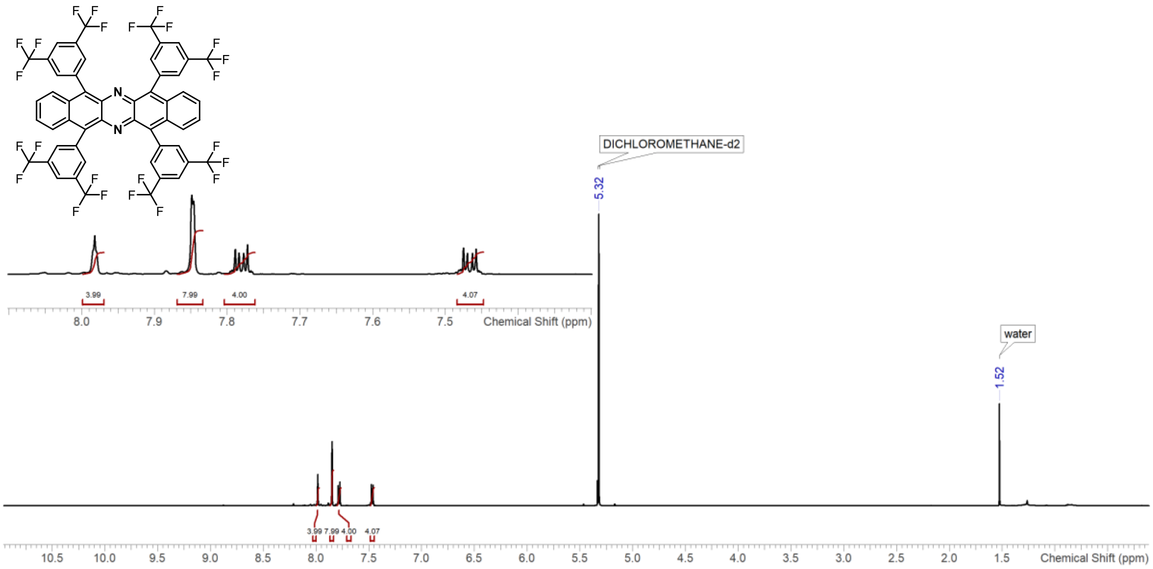

$^{13}C\{^{1}H\}$ NMR

Multiplets Integrals Sum	0.00	Number of Nuclei	0 C's

**Acquisition Time (sec)**	1.0795	**Comment**	GZ 399	**D**	0.03	**D1**	2	**DE**	18
**DS**	4	**Date**	30 Apr 2019 18:58:38			**Date Stamp**	30 Apr 2019 18:58:38		
**File Name**	\\bunz22\Mitarbeiter\Gaozhan XIE\03_analytics\01-NMR\nmr02\e190430ubgz.399\1\PDATA\1\1r					**Frequency (MHz)**	150.9314	**GB**	0
**INSTRUM**	<spect>	**LB**	1	**NS**	4096	**Nucleus**	13C	**Number of Transients**	4096
**Origin**	spect	**Original Points Count**	49066	**Owner**	ns	**PC**	1.4		
**PROBHD**	<Z132808_0001 (CP QCI 600S3 H/P/C-N-D-05 Z LT)>			**PULPROG**	<zgpg30>	**Points Count**	65536	**Pulse Sequence**	zgpg30
**Receiver Gain**	2050.00	**SF**	150.931431	**SFO1**	150.94803345741			**SI**	65536
**SSB**	0	**SW(cyclical) (Hz)**	45454.55	**SWH**	45454.5454545455				
**Solvent**	DICHLOROMETHANE-d2			**Spectrum Offset (Hz)**	16644.9277	**Spectrum Type**	standard	**Sweep Width (Hz)**	45453.85
**TD**	98132	**TD0**	512	**TE**	294.9996	**Temperature (degree C)**	22.000	**UNC1**	<13C>
**WDW**	1								

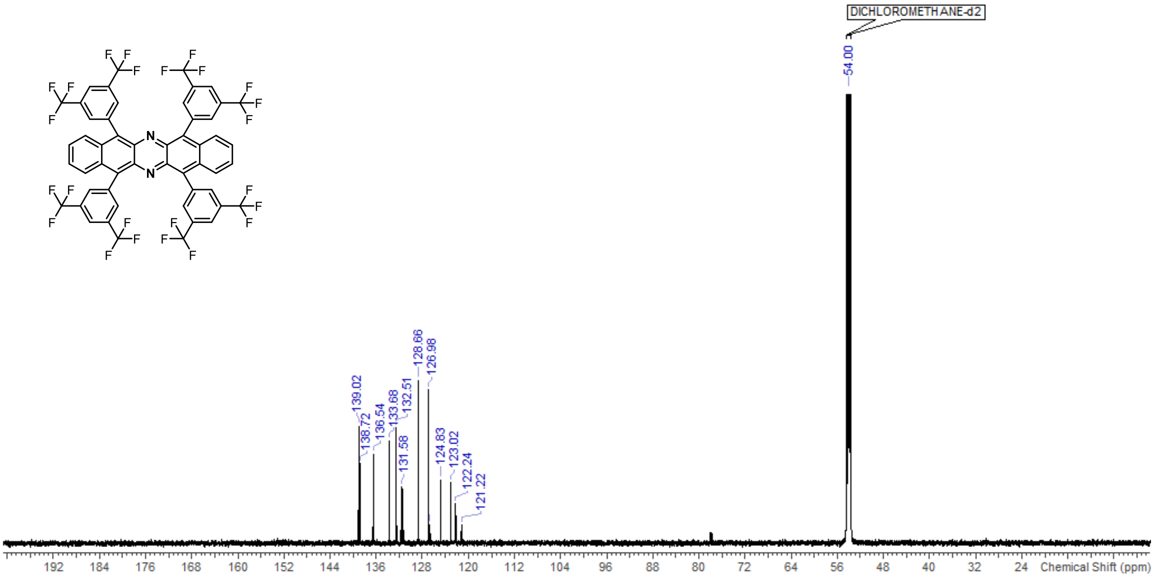

4

^{1}H NMR

Acquisition Time (sec)	2.7263	***Comment***	HH 343	***D***	0.5	***D1***	0.5	***DE***	10
DS	2	***Date***	20 Jan 2019 17:10:03			***Date Stamp***	20 Jan 2019 17:10:03		
File Name	\\bunz22\Mitarbeiter\Hoffmann\Promotion\Analytik\NMR\c190117ubhh.343\2\PDATA\1\1r					***Frequency (MHz)***	400.3300	***GB***	0
INSTRUM	<spect>	***LB***	0.3	***NS***	128	***Nucleus***	1H	***Number of Transients***	128
Origin	spect	***Original Points Count***	32768	***Owner***	ns	***PC***	1		
PROBHD	<Z130030_0004 (CPP BBO 400S1 BB-H&F-D-05 LT)>					***PULPROG***	<zg30>	***Points Count***	65536
Pulse Sequence	zg30	***Receiver Gain***	812.00	***SF***	400.33	***SFO1***	400.3320009		
SI	65536	***SSB***	0	***SW(cyclical) (Hz)***	12019.23	***SWH***	12019.2307692308		
Solvent	DMSO-d6	***Spectrum Offset (Hz)***	1997.4972	***Spectrum Type***	standard	***Sweep Width (Hz)***	12019.05	***TD***	65536
TD0	16	***TE***	295.0004	***Temperature (degree C)***	22.000	***UNC1***	<1H>	***WDW***	1

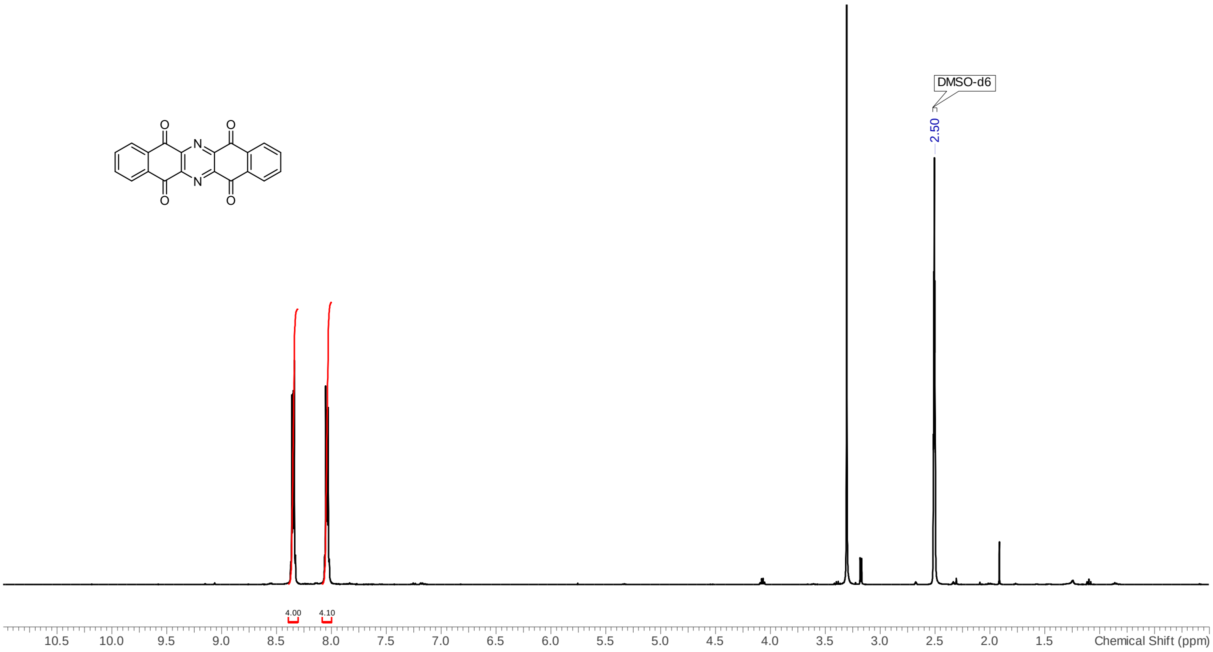

^{13}C{^{1}H} NMR

Acquisition Time (sec)	1.5897	***Comment***	HH 343	***D***	1.5	***D1***	1.5	***DE***	18
DS	2	***Date***	20 Jan 2019 17:02:12			***Date Stamp***	20 Jan 2019 17:02:12		
File Name	\\bunz22\Mitarbeiter\Hoffmann\Promotion\Analytik\NMR\c190117ubhh.343\1\PDATA\1\1r					***Frequency (MHz)***	100.6631	***GB***	0
INSTRUM	<spect>	***LB***	1	***NS***	3200	***Nucleus***	13C	***Number of Transients***	3200
Origin	spect	***Original Points Count***	49066	***Owner***	ns	***PC***	1.4		
PROBHD	<Z130030_0004 (CPP BBO 400S1 BB-H&F-D-05 LT)>					***PULPROG***	<zgpg30>	***Points Count***	65536
Pulse Sequence	zgpg30	***Receiver Gain***	2050.00	***SF***	100.663059	***SFO1***	100.67413193649		
SI	65536	***SSB***	0	***SW(cyclical) (Hz)***	30864.20	***SWH***	30864.1975308642		
Solvent	DMSO-d6	***Spectrum Offset (Hz)***	11020.9648	***Spectrum Type***	standard	***Sweep Width (Hz)***	30863.73	***TD***	98132
TD0	400	***TE***	295.0014	***Temperature (degree C)***	22.001	***UNC1***	<13C>	***WDW***	1

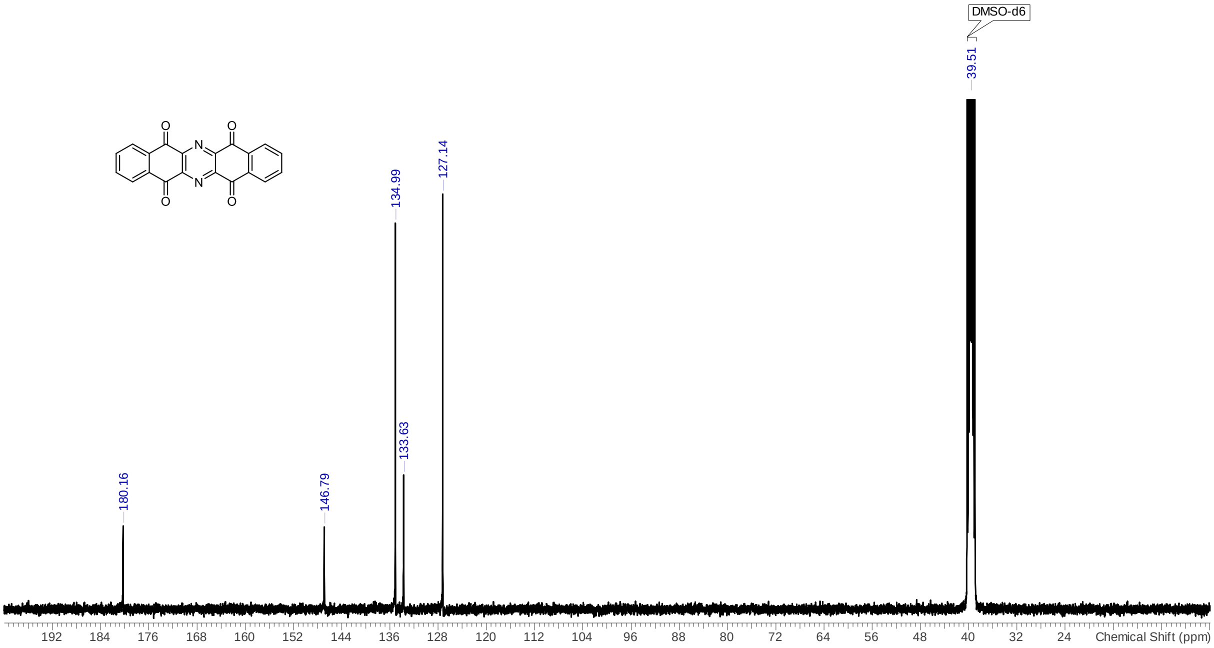

5

^{1}H NMR

Acquisition Time (sec)	3.6351	Comment	HH 345	D	0.1	D1	0.1	DE	12
DS	2	Date	19 Jan 2019 04:35:04			Date Stamp	19 Jan 2019 04:35:04		
File Name	\\bunz22\Mitarbeiter\Hoffmann\Promotion\Analytik\NMR\e190117ubhh.345\2\PDATA\1\1r					Frequency (MHz)	600.2438	GB	0
INSTRUM	<spect>	LB	0.3	NS	128	Nucleus	1H	Number of Transients	128
Origin	spect	Original Points Count	65536	Owner	ns	PC	1		
PROBHD	<Z132808_0001 (CP QCI 600S3 H/P/C-N-D-05 Z LT)>					PULPROG	<zg30>	Points Count	65536
Pulse Sequence	zg30	Receiver Gain	15.35	SF	600.243829	SFO1	600.246830219145		
SI	65536	SSB	0	SW(cyclical) (Hz)	18028.85	SWH	18028.8461538462		
Solvent	DICHLOROMETHANE-d2			Spectrum Offset (Hz)	2807.2170	Spectrum Type	standard	Sweep Width (Hz)	18028.57
TD	131072	TD0	16	TE	294.9994	Temperature (degree C)	21.999	UNC1	<1H>
WDW	1								

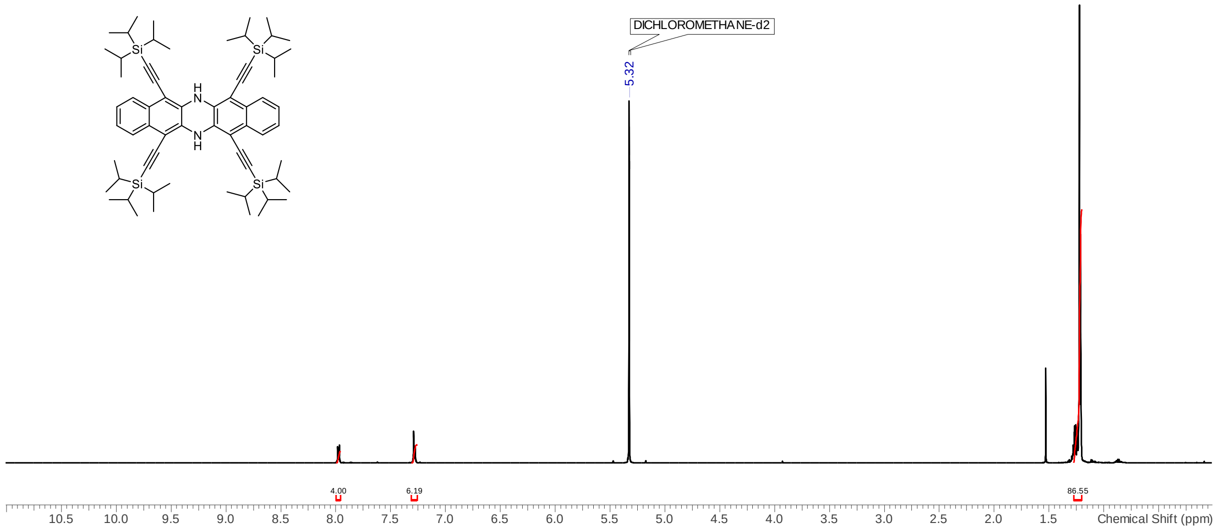

^{13}C{^{1}H} NMR

Acquisition Time (sec)	1.0795	Comment	HH 345	D	0.03	D1	2	DE	18
DS	4	Date	19 Jan 2019 04:26:00			Date Stamp	19 Jan 2019 04:26:00		
File Name	\\bunz22\Mitarbeiter\Hoffmann\Promotion\Analytik\NMR\e190117ubhh.345\1\PDATA\1\1r					Frequency (MHz)	150.9314	GB	0
INSTRUM	<spect>	LB	1	NS	4096	Nucleus	13C	Number of Transients	4096
Origin	spect	Original Points Count	49066	Owner	ns	PC	1.4		
PROBHD	<Z132808_0001 (CP QCI 600S3 H/P/C-N-D-05 Z LT)>			PULPROG	<zgpg30>	Points Count	65536	Pulse Sequence	zgpg30
Receiver Gain	2050.00	SF	150.931431	SFO1	150.94803345741			SI	65536
SSB	0	SW(cyclical) (Hz)	45454.55	SWH	45454.5454545455				
Solvent	DICHLOROMETHANE-d2			Spectrum Offset (Hz)	16644.2344	Spectrum Type	standard	Sweep Width (Hz)	45453.85
TD	98132	TD0	512	TE	295.0006	Temperature (degree C)	22.001	UNC1	<13C>
WDW	1								

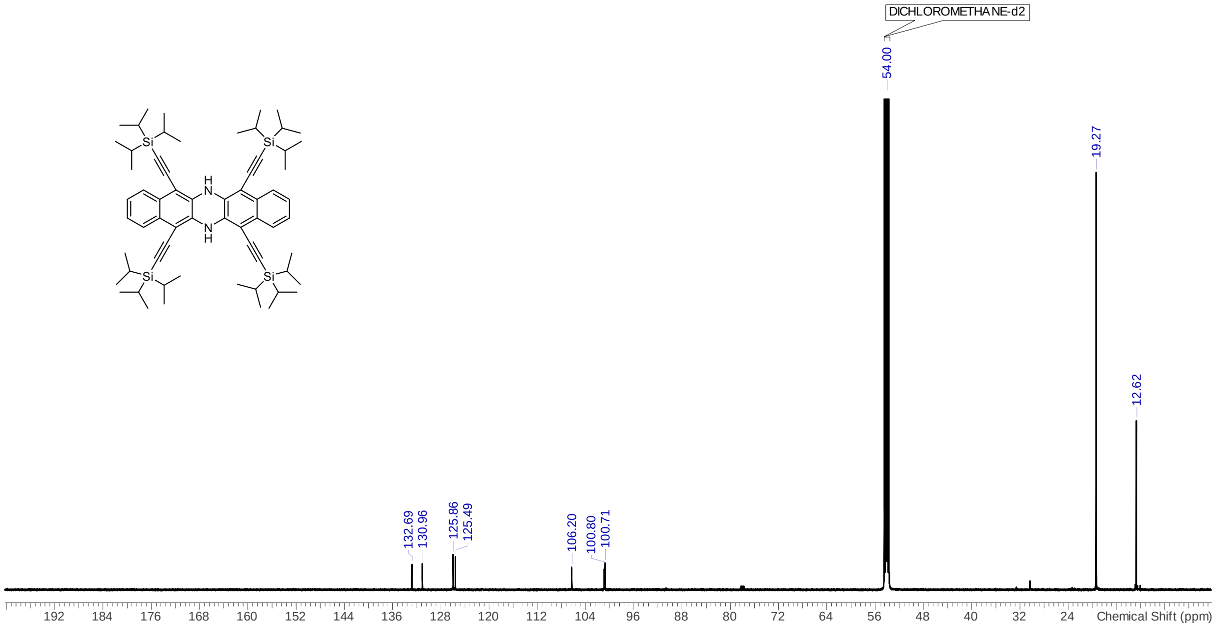

3d

^{1}H NMR

Acquisition Time (sec)	2.7263	***Comment***	HH 348-3	***D***	0.5	***D1***	0.5	***DE***	10
DS	2	***Date***	16 Jan 2019 19:31:19			***Date Stamp***	16 Jan 2019 19:31:19		
File Name	\\bunz22\Mitarbeiter\Hoffmann\Promotion\Analytik\NMR\c190116ubhh.348-3\2\PDATA\1\1r					***Frequency (MHz)***	400.3300	***GB***	0
INSTRUM	<spect>	***LB***	0.3	***NS***	128	***Nucleus***	1H	***Number of Transients***	128
Origin	spect	***Original Points Count***	32768	***Owner***	ns	***PC***	1		
PROBHD	<Z130030_0004 (CPP BBO 400S1 BB-H&F-D-05 LT)>					***PULPROG***	<zg30>	***Points Count***	65536
Pulse Sequence	zg30	***Receiver Gain***	114.00	***SF***	400.330053004643			***SFO1***	400.3320009
SI	65536	***SSB***	0	***SW(cyclical) (Hz)***	12019.23	***SWH***	12019.2307692308		
Solvent	DICHLOROMETHANE-d2			***Spectrum Offset (Hz)***	1985.5284	***Spectrum Type***	standard	***Sweep Width (Hz)***	12019.05
TD	65536	***TD0***	16	***TE***	295.0006	***Temperature (degree C)***	22.001	***UNC1***	<1H>
WDW	1								

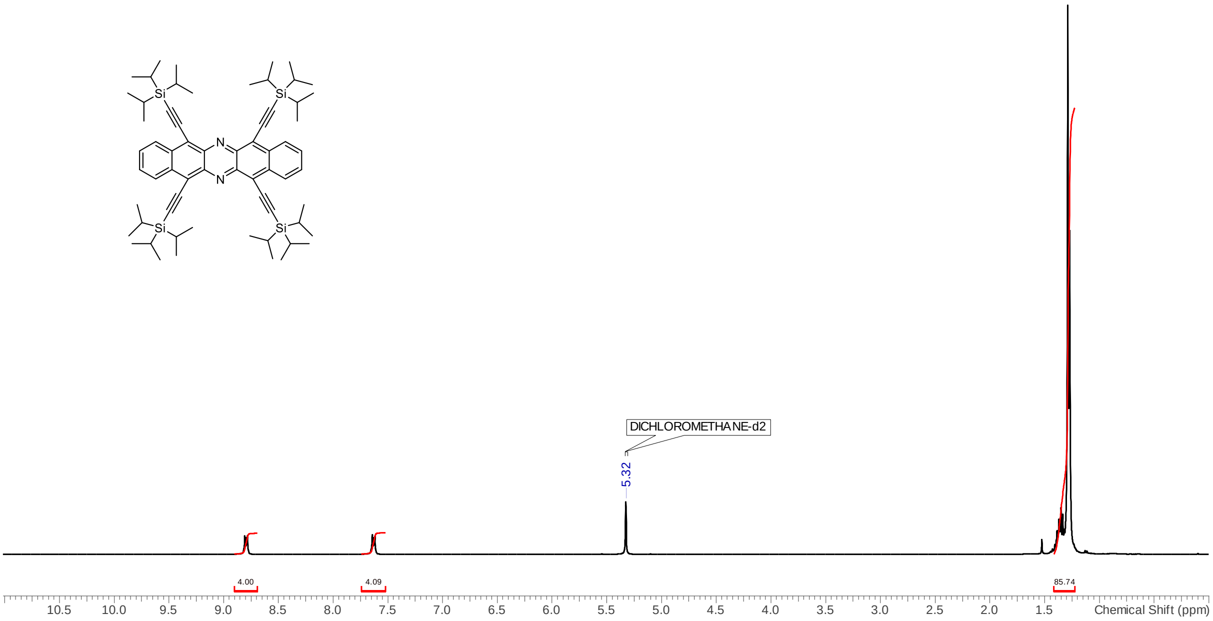

^{13}C{^{1}H} NMR

Acquisition Time (sec)	1.5897	***Comment***	HH 348-3	***D***	1.5	***D1***	1.5	***DE***	18
DS	2	***Date***	16 Jan 2019 19:23:02			***Date Stamp***	16 Jan 2019 19:23:02		
File Name	\\bunz22\Mitarbeiter\Hoffmann\Promotion\Analytik\NMR\c190116ubhh.348-3\1\PDATA\1\1r					***Frequency (MHz)***	100.6631	***GB***	0
INSTRUM	<spect>	***LB***	1	***NS***	4096	***Nucleus***	13C	***Number of Transients***	4096
Origin	spect	***Original Points Count***	49066	***Owner***	ns	***PC***	1.4		
PROBHD	<Z130030_0004 (CPP BBO 400S1 BB-H&F-D-05 LT)>			***PULPROG***	<zgpg30>	***Points Count***	65536	***Pulse Sequence***	zgpg30
Receiver Gain	2050.00	***SF***	100.663059	***SFO1***	100.67413193649			***SI***	65536
SSB	0	***SW(cyclical) (Hz)***	30864.20	***SWH***	30864.1975308642				
Solvent	DICHLOROMETHANE-d2			***Spectrum Offset (Hz)***	11135.0068	***Spectrum Type***	standard	***Sweep Width (Hz)***	30863.73
TD	98132	***TD0***	512	***TE***	294.999	***Temperature (degree C)***	21.999	***UNC1***	<13C>
WDW	1								

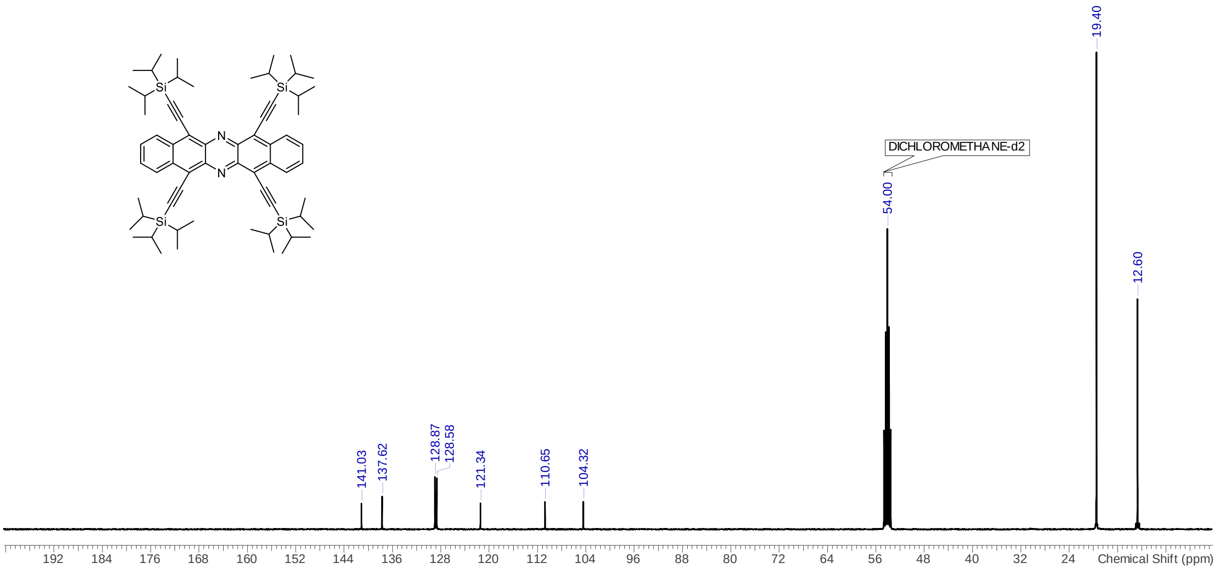

S7. Mass Spectrometry

3a

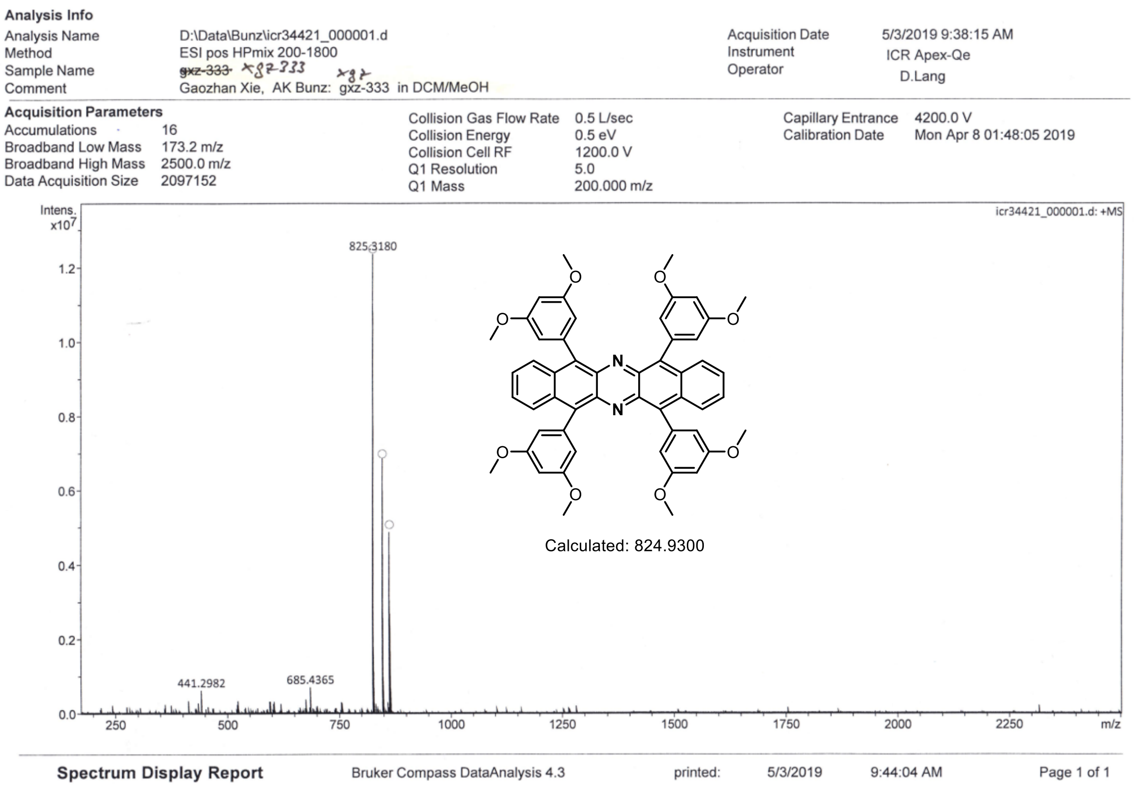

3b

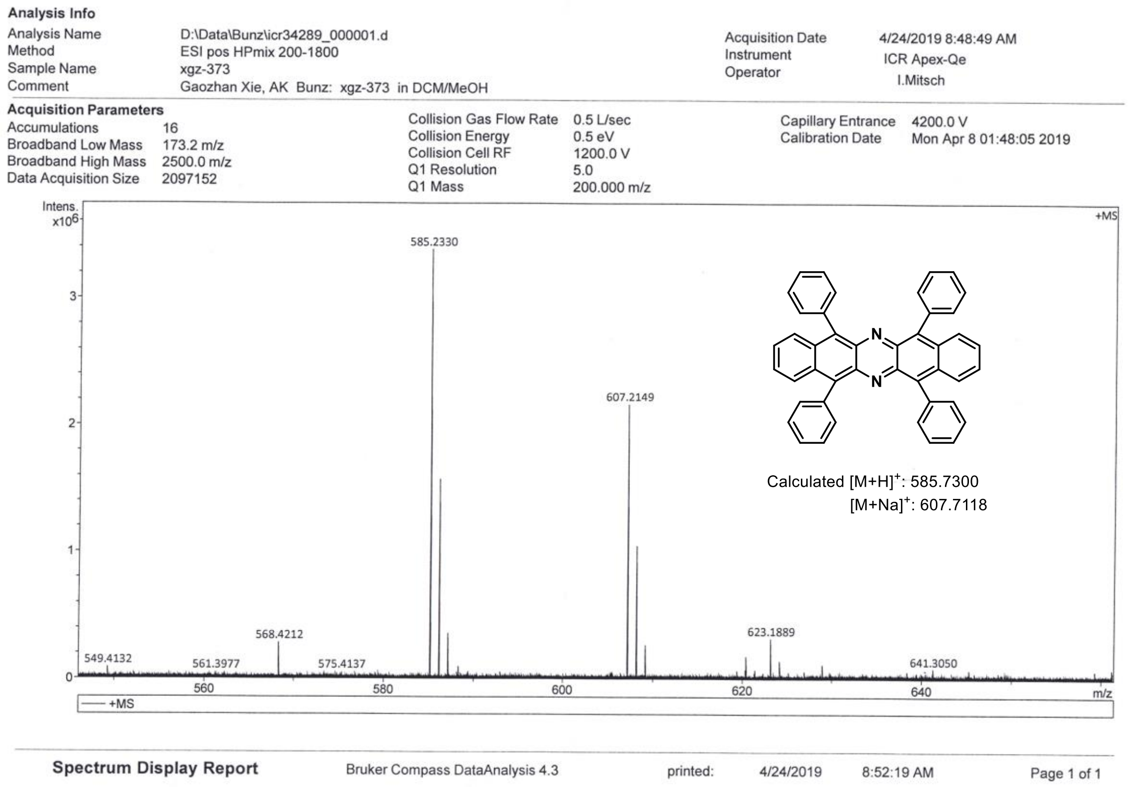

3c

Analysis Info

Analysis Name	D:\Data\Bunz\icr34592_0_G10_000001.d	Acquisition Date	5/15/2019 2:26:17 PM
Method	MALDIposCsI_300-2500_512k	Instrument	ICR Apex-Qe
Sample Name	xgz-399	Operator	I.Mitsch
Comment	Gaozhan Xie, AK Bunz: xgz-399 in DCM, Matrix: DCTB		

Acquisition Parameters

Accumulations	24	Collision Gas Flow Rate	0.8 L/sec	Laser Power	23.0 %
Broadband Low Mass	288.7 m/z	Collision Energy	0.5 eV	MALDI Plate	350.0 V
Broadband High Mass	2500.0 m/z	Collision Cell RF	1800.0 V	Calibration Date	Tue Mar 19 02:29:19 2019
Data Acquisition Size	524288	Q1 Resolution	10.0		
		Q1 Mass	200.000 m/z		

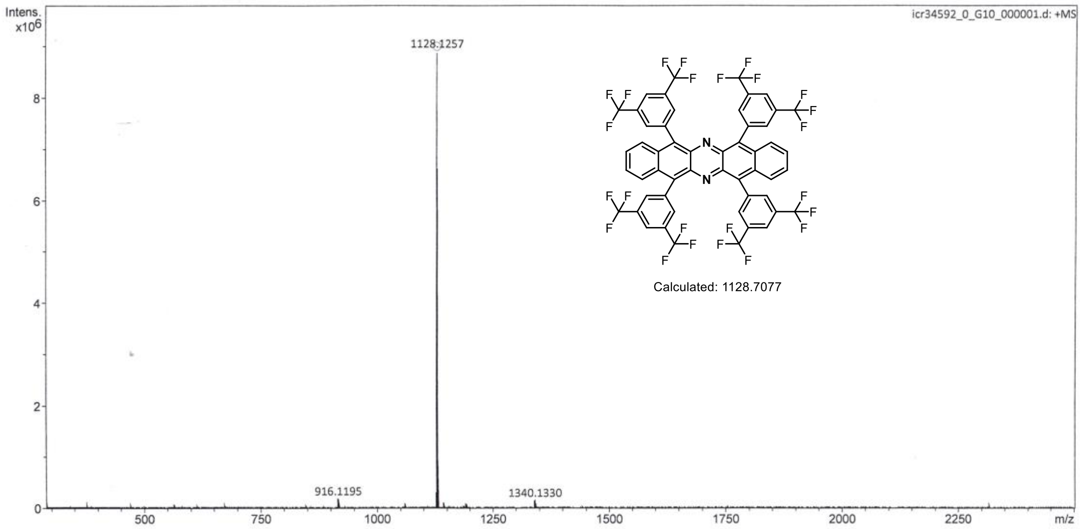

Spectrum Display Report | Bruker Compass DataAnalysis 4.3 | printed: 5/15/2019 2:29:13 PM | Page 1 of 1

4

Analysis Info

Analysis Name	D:\Data\Bunz\icr32947_000001.d	Acquisition Date	12/21/2018 9:58:02 AM
Method	DART pos IL8 200-2500	Instrument	ICR Apex-Qe DART
Sample Name	HH343	Operator	I.Mitsch
Comment	Hoffmann, AK Bunz: HH343 in DCM, 400C		

Acquisition Parameters

Broadband High Mass	2500.0 m/z	Collision Energy	0.0 eV	Calibration Date	Mon Dec 17 08:10:56 2018
Broadband Low Mass	173.2 m/z	Collision Gas Flow Rate	0.6 L/sec		
Accumulations	16	Q1 Mass	200.000 m/z		
		Q1 Resolution	7.5		

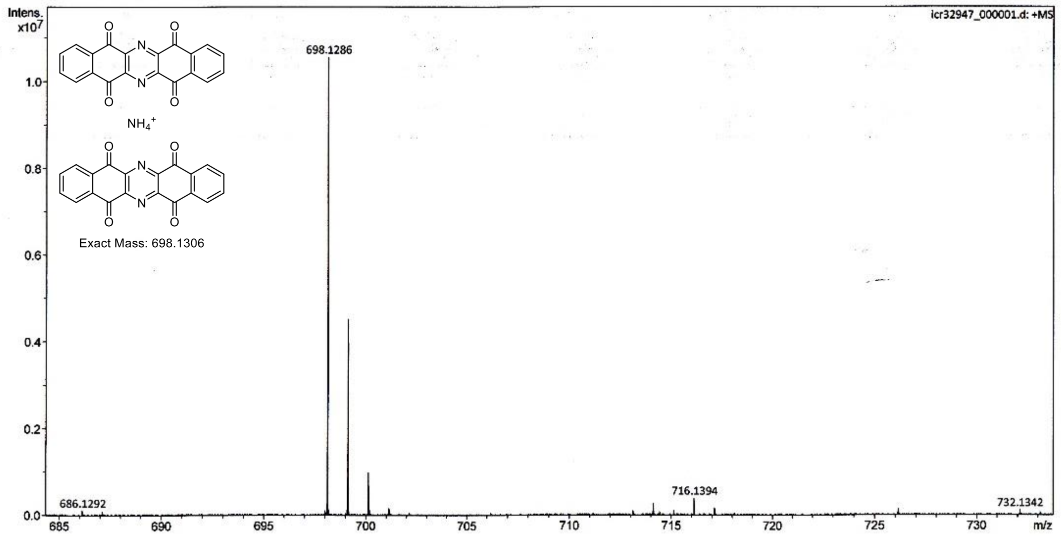

Spectrum Display Report | Bruker Compass DataAnalysis 4.3 | Page 1 of 1

5

Analysis Info

**Analysis Name**	D:\Data\Bunz\icr33145_000002.d	**Acquisition Date**	1/17/2019 12:18:05 PM
Method	DART pos IL8 200-2500	Instrument	ICR Apex-Qe DART
Sample Name	HH345	Operator	I.Mitsch
Comment	Hoffmann, AK Bunz: HH345 in DCM, 400C		

Acquisition Parameters

Broadband High Mass	2500.0 m/z	Collision Energy	0.0 eV	Calibration Date	Thu Jan 17 08:10:18 2019
Broadband Low Mass	173.2 m/z	Collision Gas Flow Rate	0.6 L/sec		
Accumulations	16	Q1 Mass	200.000 m/z		
		Q1 Resolution	7.5		

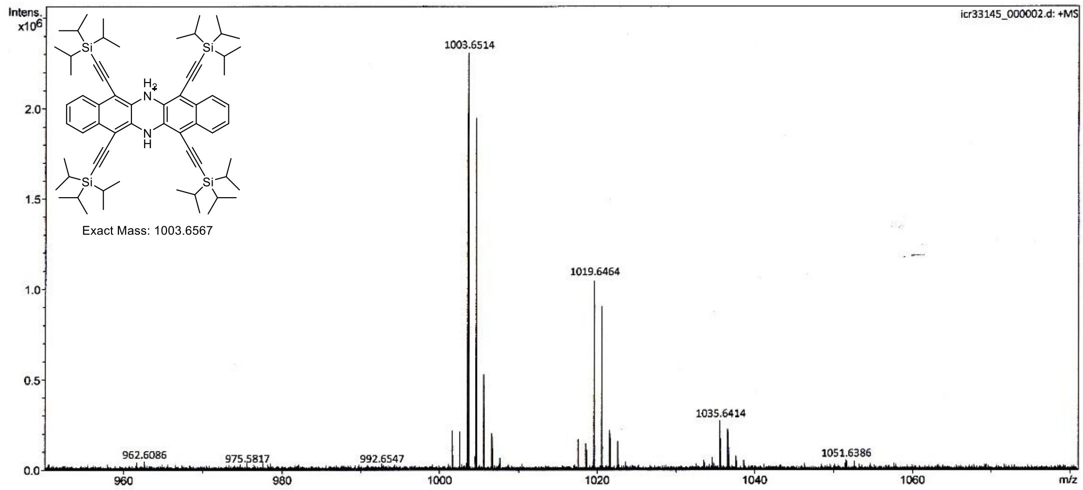

Spectrum Display Report Bruker Compass DataAnalysis 4.3 Page 1 of 1

3d

Analysis Info

**Analysis Name**	D:\Data\Bunz\icr33150_000002.d	**Acquisition Date**	1/17/2019 12:31:17 PM
Method	DART pos IL8 200-2500	Instrument	ICR Apex-Qe DART
Sample Name	HH348-3	Operator	I.Mitsch
Comment	Hoffmann, AK Bunz: HH348-3 in DCM, 400C		

Acquisition Parameters

Broadband High Mass	2500.0 m/z	Collision Energy	0.0 eV	Calibration Date	Thu Jan 17 08:10:18 2019
Broadband Low Mass	173.2 m/z	Collision Gas Flow Rate	0.6 L/sec		
Accumulations	16	Q1 Mass	200.000 m/z		
		Q1 Resolution	7.5		

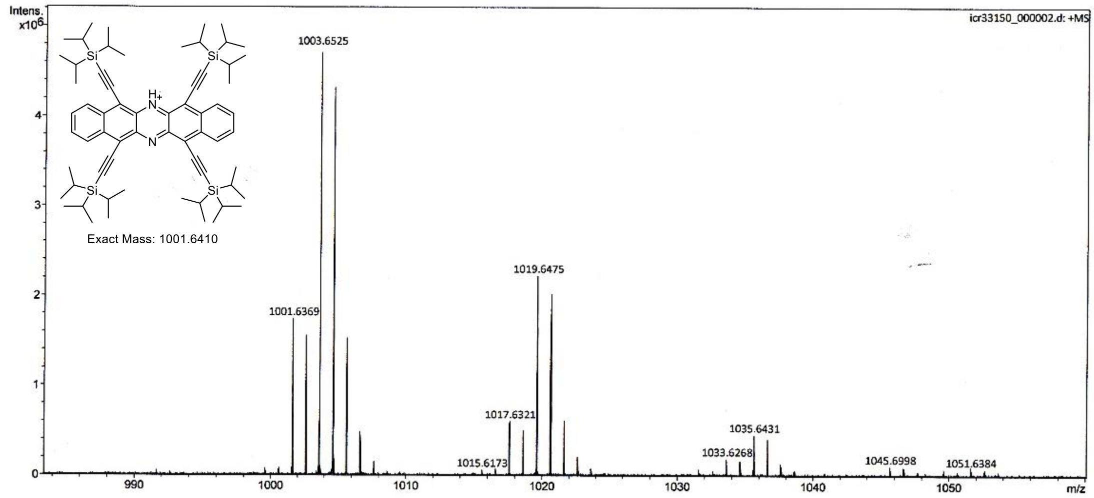

Spectrum Display Report Bruker Compass DataAnalysis 4.3 Page 1 of 1

S8. Infrared Spectroscopy

3a

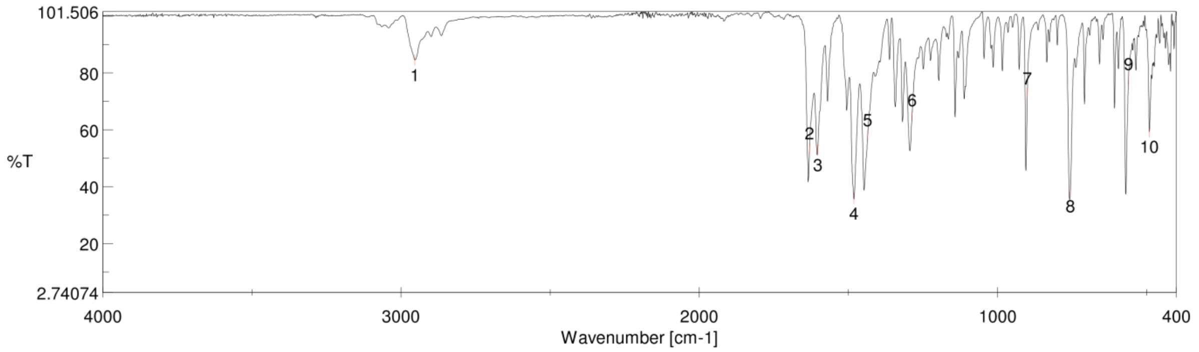

[Result of Peak Picking]

No.	Position	Intensity	No.	Position	Intensity	No.	Position	Intensity
1	2953.45	84.5058	2	1630.04	53.3593	3	1602.56	52.784
4	1481.54	35.7649	5	1434.78	57.9652	6	1286.29	64.9186
7	899.63	72.5863	8	755.477	38.3379	9	560.22	77.7554
10	490.313	59.2333						

3b

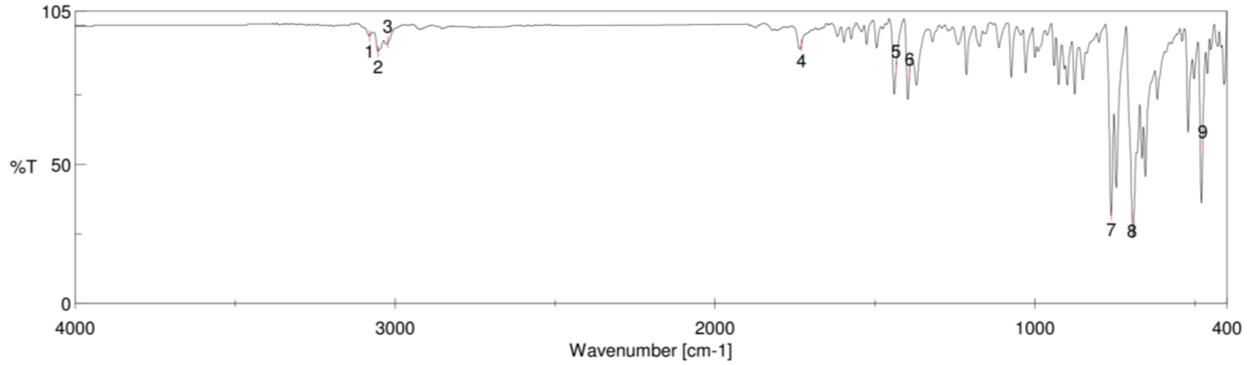

[Result of Peak Picking]

No.	Position	Intensity	No.	Position	Intensity	No.	Position	Intensity
1	3080.24	96.0565	2	3053.25	90.5514	3	3022.87	93.8289
4	1730.32	92.8051	5	1433.82	84.7	6	1392.35	82.0203
7	761.262	31.981	8	696.659	31.6923	9	475.849	55.9761

3c

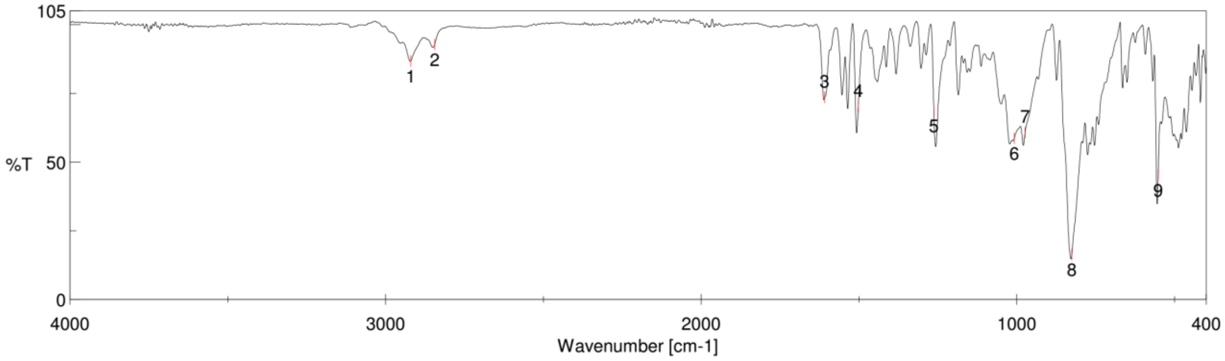

[Result of Peak Picking]

No.	Position	Intensity	No.	Position	Intensity	No.	Position	Intensity
1	2920.18	86.7043	2	2844.49	92.7339	3	1608.82	73.5095
4	1502.28	70.1409	5	1263.15	68.6691	6	1008.11	58.5912
7	973.876	60.6949	8	825.866	16.2956	9	552.024	45.2522

S9. Crystals Structures

The single crystals of **3a** and **3c** were cultivated by slow evaporation of THF and DCM respectively in glovebox under N_2. The single crystal of **3b** was cultivated by slow evaporation of DCM under ambient conditions.

Crystallographic data: CCDC 1957020 (**3a**), 1957021 (**3c**) and 1957022 (**3d**) contain the supplementary crystallographic data for this paper. The data can be obtained free of charge from The Cambridge Crystallographic Data Centre via www.ccdc.cam.ac.uk/structures.

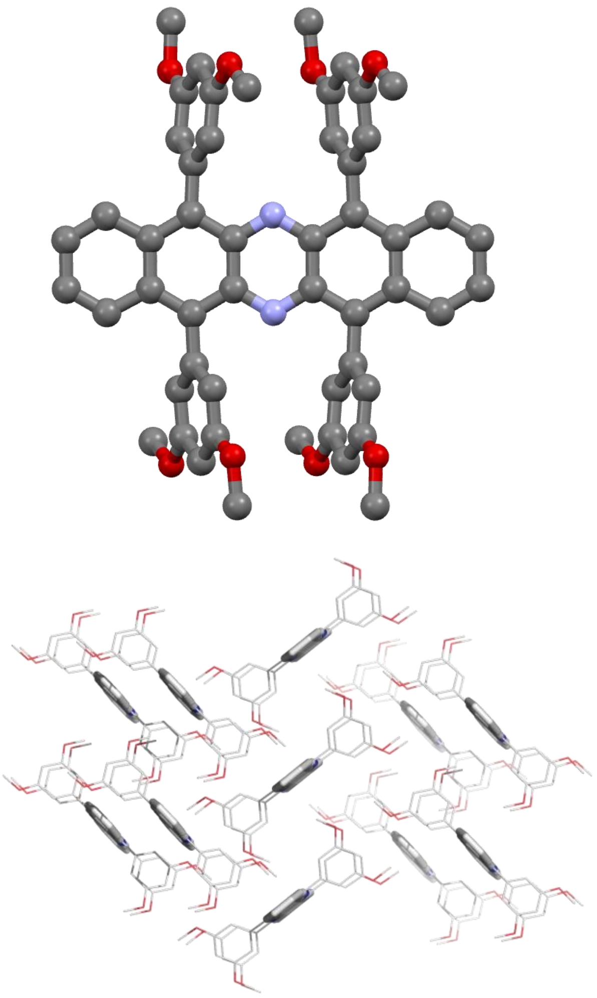

Figure S4. Molecular structure and solid-state packing of **3a**.

3a

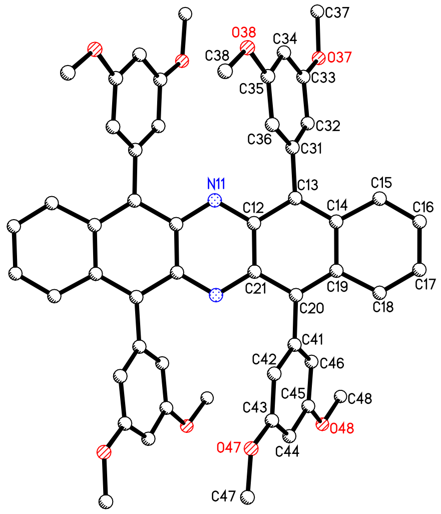

CCDC	1957020	
Empirical formula	$C_{60}H_{60}N_2O_{10}$	
Formula weight	969.10	
Temperature	110(2) K	
Wavelength	1.54178 Å	
Crystal system	monoclinic	
Space group	$P2_1/n$	
Z	2	
Unit cell dimensions	a = 12.0746(6) Å	α = 90 deg.
	b =8.6963(3) Å	β =101.040(4) deg.
	c = 24.0293(12) Å	γ = 90 deg.
Volume	2476.5(2) $Å^3$	
Density (calculated)	1.30 g/cm^3	
Absorption coefficient	0.71 mm^{-1}	
Crystal shape	brick	
Crystal size	0.162 x 0.065 x 0.057 mm^3	
Crystal colour	green	
Theta range for data collection	4.5 to 62.1 deg.	
Index ranges	$-13 \le h \le 12$, $-9 \le k \le 9$, $-27 \le l \le 16$	
Reflections collected	12244	
Independent reflections	3763 (R(int) = 0.0196)	
Observed reflections	3214 ($I > 2\sigma(I)$)	
Absorption correction	Semi-empirical from equivalents	
Max. and min. transmission	2.21 and 0.57	
Refinement method	Full-matrix least-squares on F^2	
Data/restraints/parameters	3763 / 236 / 375	
Goodness-of-fit on F^2	1.03	
Final R indices (I>2sigma(I))	R1 = 0.037, wR2 = 0.094	
Largest diff. peak and hole	0.25 and -0.27 $eÅ^{-3}$	

3b

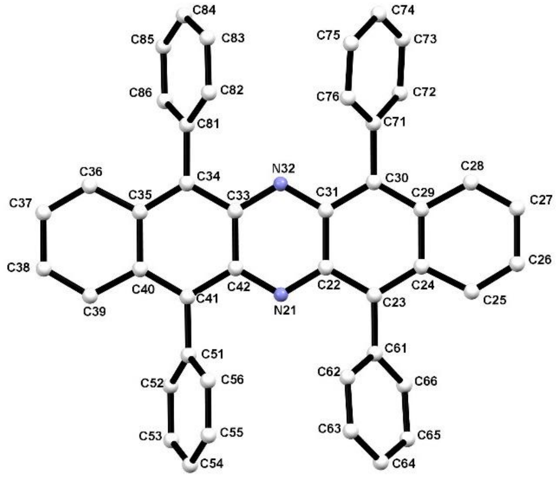

CCDC	1958306	
Empirical formula	$C_{44}H_{28}N_2$	
Formula weight	584.68	
Temperature	153 K	
Wavelength	1.54184 Å	
Crystal system	triclinic	
Space group	P-1	
Z	2	
Unit cell dimensions	a = 9.8329(14) Å	α = 100.143(11) deg.
	b =13.3353(18) Å	β = 105.860(12) deg.
	c = 14.1833(18) Å	γ = 108.504(13) deg.
Volume	1624.5(4) $Å^3$	
Density (calculated)	1.195 g/cm^3	
Absorption coefficient	0.532 mm^{-1}	
Crystal shape	plank	
Crystal size	0.238 x 0.095 x 0.037 mm^3	
Crystal colour	green	
Theta range for data collection	7.3 to 146.004 deg.	
Index ranges	$-9 \leq h \leq 12$, $-16 \leq k \leq 12$, $-17 \leq l \leq 16$	
Reflections collected	9375	
Independent reflections	6073 (R(int) = 0.0598)	
Observed reflections	3318 (I > 2σ(I))	
Absorption correction	Semi-empirical from equivalents	
Max. and min. transmission	1.00 and 0.86	
Refinement method	Full-matrix least-squares on F^2	
Data/restraints/parameters	6073 / 0 / 415	
Goodness-of-fit on F^2	0.976	
Final R indices (I>2sigma(I))	R1 = 0.0626, wR2 = 0.1790	
Largest diff. peak and hole	0.25 and -0.21 $eÅ^{-3}$	

3c

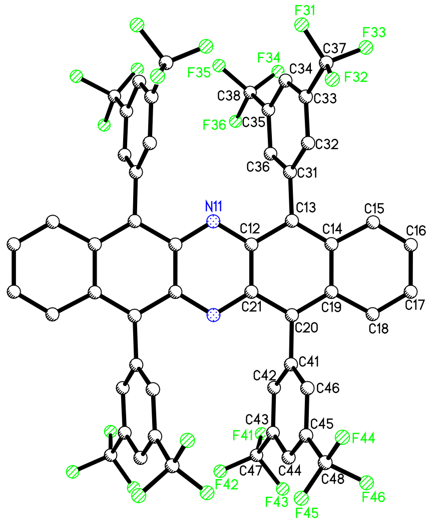

CCDC	1957021	
Empirical formula	$C_{52}H_{20}F_{24}N_2$	
Formula weight	1128.70	
Temperature	200(2) K	
Wavelength	1.54178 Å	
Crystal system	monoclinic	
Space group	$P2_1/n$	
Z	2	
Unit cell dimensions	a = 12.859(3) Å	α = 90 deg.
	b =7.9086(16) Å	β = 103.83(3) deg.
	c = 23.298(5) Å	γ = 90 deg.
Volume	2300.8(8) $Å^3$	
Density (calculated)	1.63 g/cm^3	
Absorption coefficient	1.48 mm^{-1}	
Crystal shape	plank	
Crystal size	0.126 x 0.041 x 0.015 mm^3	
Crystal colour	blue	
Theta range for data collection	3.9 to 57.9 deg.	
Index ranges	$-12\leq h\leq 14$, $-6\leq k\leq 8$, $-25\leq l\leq 24$	
Reflections collected	11891	
Independent reflections	3181 (R(int) = 0.0731)	
Observed reflections	1842 ($I > 2\sigma(I)$)	
Absorption correction	Semi-empirical from equivalents	
Max. and min. transmission	1.49 and 0.61	
Refinement method	Full-matrix least-squares on F^2	
Data/restraints/parameters	3181 / 1316 / 464	
Goodness-of-fit on F^2	1.04	
Final R indices (I>2sigma(I))	R1 = 0.053, wR2 = 0.097	
Largest diff. peak and hole	0.19 and -0.24 $eÅ^{-3}$	

3d

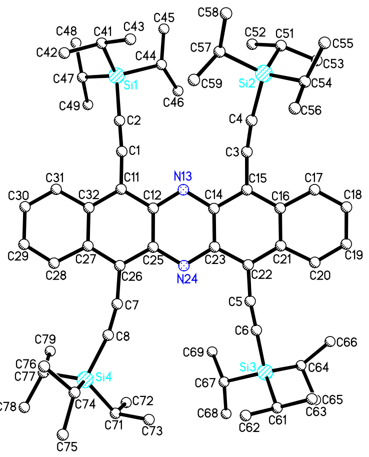

CCDC	1957022	
Empirical formula	$C_{64}H_{92}N_2Si_4$	
Formula weight	1001.75	
Temperature	200(2) K	
Wavelength	0.71073 Å	
Crystal system	monoclinic	
Space group	C2/c	
Z	8	
Unit cell dimensions	a = 54.797(5) Å	α = 90 deg.
	b = 14.9362(14) Å	β =94.8650(16) deg.
	c = 15.1967(14) Å	γ = 90 deg.
Volume	12393(2) Å^3	
Density (calculated)	1.07 g/cm^3	
Absorption coefficient	0.13 mm^{-1}	
Crystal shape	lamina	
Crystal size	0.200 x 0.190 x 0.100 mm^3	
Crystal colour	blue	
Theta range for data collection	0.7 to 28.8 deg.	
Index ranges	$-74 \le h \le 73$, $-20 \le k \le 20$, $-20 \le l \le 20$	
Reflections collected	150303	
Independent reflections	16072 (R(int) = 0.0794)	
Observed reflections	11858 ($I > 2\sigma(I)$)	
Absorption correction	Semi-empirical from equivalents	
Max. and min. transmission	0.96 and 0.62	
Refinement method	Full-matrix least-squares on F^2	
Data/restraints/parameters	16072 / 0 / 655	
Goodness-of-fit on F^2	1.03	
Final R indices (I>2sigma(I))	R1 = 0.050, wR2 = 0.125	
Largest diff. peak and hole	0.42 and -0.27 eÅ^{-3}	

S10 Device Fabrication

Substrate modification:

Si/SiO_2 substrates (300 nm) were modified by self-assembled monolayer of octadecyltrichlorosilane (OTS). Prior to functionalization substrates were cleaned in piranha solution. The process was carried out in a mixture of chloroform and *n*-hexane in a ratio of 1 : 4 and in the presence of OTS in relation to the volume of the mixture of 1 : 200. The substrates were kept in the solution for 30 minutes and then rinsed three times with chloroform in an ultrasonic cleaner and then dried under a nitrogen flux. Solvents (AR grade) and OTS were purchased from Sigma-Aldrich and used without further purification.

Thin film deposition:

Both materials were deposited in the same conditions. Thin films of 50 nm (indication of quartz weight calibrated on pentacene) were thermally evaporated under high vacuum with the growth rate of 0.1 Å s^{-1} and substrate temperature of 60 °C. Process was carried out in MBraun MB-ProVap-7 evaporation chamber (BioNanoPark in Lodz, Poland).

Electrodes deposition:

Transistors were prepared in top-contact / bottom gate configuration. The 50 nm thick source and drain electrodes were thermally evaporated under high vacuum with the growth rate of 1.0 nm s^{-1}. Silver electrodes were used. Ossila E325 Linear shadow source-drain masks with variable channel length from 10 µm to 30 µm and channel width of 1 mm were used.

Field effect measurements:

OFET measurements were performed by using a Keithley 2634B source meter. Transfer characteristics were measured under a nitrogen atmosphere in the range of V_{GS} from 0 V to 80 V. The same range of changes was used for V_{DS} during output characteristics measurements. The charge carrier mobility was derived from transfer characteristics ($|V_{DS}$ = 80V$|$) in the saturation regime. For each sample twelve transistors were measured.

S11. References

[1] G. W. T. M. J. Frisch, H. B. Schlegel, G. E. Scuseria, M. A. Robb, J. R. Cheeseman, I. G. Scalmani, V. Barone, B. Mennucci, G. A. Petersson, H. Nakatsuji, M. Caricato, X. Li, H. P. Hratchian, A. F. Izmaylov, J. Bloino, G. Zheng, J. L. Sonnenberg, M. Hada, M. Ehara, K. Toyota, R. Fukuda, J. Hasegawa, M. Ishida, T. Nakajima, Y. Honda, O. Kitao, H. Nakai, T. Vreven, J. A., Jr., Montgomery, J. E. Peralta, F. Ogliaro, M. J. Bearpark, J. Heyd, E. N. Brothers, K. Kudin, V. N. Staroverov, R. Kobayashi, J. Normand, K. Raghavachari, A. P. Rendell, J. C. Burant, S. S. Iyengar, J. Tomasi, M. Cossi, N. Rega, N. J. Millam, M. Klene, J. E. Knox, J. B. Cross, V. Bakken, C. Adamo, J. Jaramillo, R. Gomperts, R. E. Stratmann, O. Yazyev, A. J. Austin, R. Cammi, C. Pomelli, J. W. Ochterski, R. L. Martin, K. Morokuma, V. G. Zakrzewski, G. A. Voth, P. Salvador, J. J. Dannenberg, S. Dapprich, A. D. Daniels, O. Farkas, J. B. Foresman, J. V. Ortiz, D. J. Cioslowski, D. Fox, J., Gaussian09, Wallingford, **2009**.

[2] C. M. Cardona, W. Li, A. E. Kaifer, D. Stockdale, G. C. Bazan, *Adv. Mater.* **2011**, 23, 2367-2371.

S12. Cartesian Coordinates of computational studied molecules

Coordinates of **3a**:

C	-0.71537400	6.04108200	-0.00007600
C	0.71533300	6.04108700	-0.00019200
C	1.40760900	4.86336200	-0.00017500
C	0.73012400	3.59197800	-0.00004800
C	-0.73014400	3.59197400	0.00004000
C	-1.40764000	4.86335100	0.00003500
C	1.44353300	2.38701600	-0.00001100
C	0.72892400	1.15402800	0.00011500
C	-0.72892700	1.15402400	0.00015900
C	-1.44354500	2.38700600	0.00013100
N	1.41430000	0.00000700	0.00013500
C	0.72893100	-1.15401800	0.00016400
C	-0.72892000	-1.15402100	0.00012000
N	-1.41429500	-0.00000100	0.00013600
C	1.44354700	-2.38700200	0.00013500
C	0.73014400	-3.59196700	0.00004100
C	-0.73012300	-3.59197200	-0.00004500
C	-1.44353100	-2.38700800	-0.00000400
C	1.40764000	-4.86334500	0.00003600
C	0.71537300	-6.04107500	-0.00007800
C	-0.71533400	-6.04108100	-0.00019800
C	-1.40761000	-4.86335600	-0.00017800
C	-2.94016900	2.37885400	0.00019600
C	2.94015800	2.37887500	-0.00012900
C	-2.94015600	-2.37885700	-0.00011400
C	2.94017200	-2.37886100	0.00020200

C	3.63821500	-2.39481400	-1.21110800
C	5.03557300	-2.42895200	-1.20560700
C	5.75178600	-2.44940600	0.00035000
C	5.03544600	-2.42887200	1.20623400
C	3.63809100	-2.39470300	1.21158500
C	3.63804000	2.39451700	-1.21153700
C	5.03539600	2.42865500	-1.20623200
C	5.75177500	2.44938700	-0.00037700
C	5.03559700	2.42915600	1.20560700
C	3.63824200	2.39501400	1.21115700
C	-3.63821100	2.39473000	-1.21111900
C	-5.03556600	2.42884500	-1.20562600
C	-5.75178600	2.44934300	0.00033000
C	-5.03545000	2.42887600	1.20621200
C	-3.63809200	2.39473700	1.21157400
C	-3.63804800	-2.39443500	-1.21151700
C	-5.03540400	-2.42855600	-1.20620300
C	-5.75177400	-2.44933000	-0.00034400
C	-5.03558700	-2.42915700	1.20563600
C	-3.63823100	-2.39503600	1.21117600
H	-1.25140400	6.98584100	-0.00007900
H	1.25135600	6.98585000	-0.00029400
H	2.49122500	4.86760800	-0.00025900
H	-2.49125600	4.86758600	0.00011900
H	2.49125600	-4.86758200	0.00012100
H	1.25140400	-6.98583300	-0.00008100
H	-1.25135600	-6.98584400	-0.00030600
H	-2.49122500	-4.86759900	-0.00026700
H	3.11606900	-2.37983900	-2.16122200
H	6.83185100	-2.47909100	0.00040600
H	3.11584600	-2.37963400	2.16164400
H	3.11576500	2.37930100	-2.16157700
H	6.83184200	2.47897200	-0.00047500
H	3.11612000	2.38019500	2.16128700
H	-3.11606000	2.37970700	-2.16123000
H	-6.83185100	2.47903300	0.00038200
H	-3.11585400	2.37972200	2.16163800
H	-3.11578000	-2.37918000	-2.16156100
H	-6.83184000	-2.47893400	-0.00043500
H	-3.11610300	-2.38026000	2.16130300
O	-5.63121500	-2.44793900	-2.44001400
O	-5.63158100	-2.44917100	2.43935100
O	-5.63153300	2.44854300	-2.43935700
O	-5.63129300	2.44865600	2.43999500
O	5.63119600	2.44810500	-2.44004700
O	5.63160300	2.44909600	2.43931700

O	5.63128100	-2.44857600	2.44002600
O	5.63154200	-2.44871900	-2.43933400
C	7.04685600	2.51102900	2.51774500
H	7.28591200	2.52342700	3.58218900
H	7.51582500	1.63392900	2.05250300
H	7.43741400	3.42365700	2.04857900
C	7.04645400	2.50961900	-2.51872100
H	7.51525400	1.63263000	-2.05310600
H	7.28534800	2.52142300	-3.58320800
H	7.43732900	3.42238400	-2.05008100
C	-7.04655900	2.50996600	2.51859000
H	-7.51519900	1.63286100	2.05302400
H	-7.28551300	2.52182800	3.58306300
H	-7.43755400	3.42262400	2.04984600
C	-7.04679400	2.51018500	-2.51783600
H	-7.28582800	2.52221800	-3.58228900
H	-7.51560700	1.63313800	-2.05233800
H	-7.43753600	3.42288300	-2.04895600
C	7.04653900	-2.51008100	2.51866100
H	7.51531900	-1.63302800	2.05314300
H	7.28545900	-2.52200900	3.58314000
H	7.43741900	-3.42278200	2.04989900
C	7.04680000	-2.51044000	-2.51779800
H	7.28584200	-2.52255100	-3.58224800
H	7.51565200	-1.63338500	-2.05235100
H	7.43749100	-3.42312500	-2.04885500
C	-7.04684900	-2.51073500	2.51779000
H	-7.28590300	-2.52298600	3.58223600
H	-7.51559100	-1.63355400	2.05247500
H	-7.43764800	-3.42330200	2.04870400
C	-7.04646700	-2.50959200	-2.51868200
H	-7.51535500	-1.63264900	-2.05306500
H	-7.28536600	-2.52141500	-3.58316700
H	-7.43724800	-3.42239400	-2.05003800

Coordinates of **3b**:

N	2.81697	2.54878	5.56650
C	2.67502	3.48926	6.50494
C	3.23385	4.78125	6.29198
C	3.04222	5.77739	7,26400
C	3.64211	7.08424	7.15295
H	4.14544	7.28491	6.39781
C	3.49697	8.02906	8.11060
H	-5.94473	8.86406	7.99171
C	2.76135	7.76426	9.28904
H	2.66774	8.42003	9.94227

C	2.19384	6.54507	9.45757
H	1.71595	6.37993	10.23754
C	2.30116	5.49700	8.48163
C	1.76979	4.22375	8.70374
C	1.94016	3.21196	7.72911
N	1.39492	2.00326	7.93422
C	1.57058	1.04442	7.01054
C	1.01572	-0.24923	7.22481
C	1.23247	-1.24502	6.26847
C	-3.40828	10.03760	6.49971
H	-3.82254	9.82327	7.30450
C	-3.21031	9.05705	5.58518
H	-3.49075	8.18840	5.76547
C	-2.57347	9.35607	4.35187
H	-2.46522	8.68633	3.71561
C	-2.11791	10.61290	4.09188
H	-1.68979	10.78162	3.28317
C	1.95037	-0.95612	5.03515
C	2.46181	0.32215	4.79215
C	2.30360	1.33310	5.78638
C	3.20225	0.63300	3.54055
C	2.58341	0.55958	2.29678
H	1.68845	0.31519	2.24322
C	3.28724	0.84496	1.13402
H	2.86231	0.79749	0.30833
C	4.62509	1.20280	1.20980
H	5.10299	1.38398	0.43114
C	5.24742	1.28922	2.43135
H	6.14422	1.53139	2.47969
C	4.54157	1.01435	3.59281
H	4.96615	1.08497	4.41719
C	4.06737	5.05968	5.08088
C	5.21563	4.30114	4.81959
H	5.42841	3.58088	5.36831
C	6.03651	4.61591	3.74828
H	6.80247	4.11203	3.58627
C	5.72069	5.67653	2.91736
H	6.27532	5.89030	2.20272
C	4.57365	6.41977	3.15383
H	4.36154	7.13006	2.59205
C	3.74019	6.11338	4.22253
H	2.96602	6.60925	4.36493
C	1.11078	3.90533	10.01413
C	-0.01567	4.58629	10.44919
H	-0.42102	5.20979	9.89001
C	-0.54433	4.35022	11.70863

H	-1.29815	4.81754	11.99213
C	0.04233	3.42949	12.53693
H	3.57195	7.21135	0.32139
C	1.16089	2.71613	12.10449
H	1.55240	2.08678	12.66627
C	1.69262	2.93868	10.84374
H	2.42868	2.45008	10.55109
C	0.21812	-0.51877	8.45681
C	0.75227	-0.34398	9.72932
H	1.62200	-0.02666	9.82208
C	-0.00046	-0.63746	10.86856
H	0.36783	-0.51749	11.71385
C	-1.28799	-1.10398	10.73792
H	-1.77687	-1.32606	11.49698
C	-1.86272	-1.24547	9.48501
H	-2.74360	-1.53305	9.40270
C	-1.10932	-0.95570	8.34968
H	-1.49233	-1.05243	7.50700

Coordinates of **3c**:

C	0.71525425	6.04178128	0.00000000
C	-0.71525752	6.04178149	0.00000000
C	-1.40762686	4.86385170	0.00000000
C	-0.73043404	3.59243806	0.00000000
C	0.73043241	3.59243817	0.00000000
C	1.40762319	4.86385124	0.00000000
C	-1.44488692	2.38742093	0.00000000
C	-0.72877965	1.15468202	0.00000000
C	0.72877964	1.15468527	0.00000000
C	1.44488546	2.38741888	0.00000000
N	-1.41357380	0.00000116	0.00000000
C	-0.72877965	-1.15468529	0.00000000
C	0.72877944	-1.15468203	0.00000000
N	1.41357369	-0.00000084	0.00000000
C	-1.44488543	-2.38741863	0.00000000
C	-0.73043224	-3.59243808	0.00000000
C	0.73043409	-3.59243793	0.00000000
C	1.44488686	-2.38742051	0.00000000
C	-1.40762303	-4.86385102	0.00000000
C	-0.71525407	-6.04178112	0.00000000
C	0.71525757	-6.04178133	0.00000000
C	1.40762692	-4.86385145	0.00000000
C	2.94052764	2.37318944	0.00000000

C	-2.94052830	2.37319315	0.00000000
C	2.94052821	-2.37319265	0.00000000
C	-2.94052762	-2.37318960	0.00000000
C	-3.65455377	-2.37717257	1.20677199
C	-5.05127246	-2.38987768	1.20757874
C	-5.75413550	-2.39757108	0.00000000
C	-5.05127246	-2.38987768	-1.20757874
C	-3.65455377	-2.37717257	-1.20677199
C	-3.65455387	2.37719123	1.20677198
C	-5.05127205	2.38990895	1.20757875
C	-5.75413513	2.39759440	0.00000000
C	-5.05127205	2.38990895	-1.20757875
C	-3.65455387	2.37719123	-1.20677198
C	3.65455379	2.37717214	1.20677197
C	5.05127248	2.38987655	1.20757873
C	5.75413550	2.39756941	0.00000000
C	5.05127248	2.38987655	-1.20757873
C	3.65455379	2.37717214	-1.20677197
C	3.65455378	-2.37719068	1.20677196
C	5.05127196	-2.38990843	1.20757875
C	5.75413508	-2.39759381	0.00000000
C	5.05127196	-2.38990843	-1.20757875
C	3.65455378	-2.37719068	-1.20677196
H	1.25131013	6.98642659	0.00000000
H	-1.25131206	6.98642744	0.00000000
H	-2.49136984	4.86912654	0.00000000
H	2.49136593	4.86913061	0.00000000
H	-2.49136576	-4.86913044	0.00000000
H	-1.25131001	-6.98642638	0.00000000
H	1.25131219	-6.98642723	0.00000000
H	2.49136986	-4.86912642	0.00000000
H	-3.11160388	-2.36964006	2.14778476
H	-5.58903606	-2.39169396	2.15161287
H	-6.84036319	-2.40621079	0.00000000
H	-5.58903606	-2.39169396	-2.15161287
H	-3.11160388	-2.36964006	-2.14778476
H	-3.11160361	2.36965626	2.14778449
H	-5.58903527	2.39173666	2.15161298
H	-6.84036261	2.40625849	0.00000000
H	-5.58903527	2.39173666	-2.15161298
H	-3.11160361	2.36965626	-2.14778449
H	3.11160391	2.36963996	2.14778474
H	5.58903612	2.39169258	2.15161286
H	6.84036325	2.40620776	0.00000000
H	5.58903612	2.39169258	-2.15161286
H	3.11160391	2.36963996	-2.14778474

H	3.11160352	-2.36965558	2.14778446
H	5.58903522	-2.39173612	2.15161298
H	6.84036256	-2.40625739	0.00000000
H	5.58903522	-2.39173612	-2.15161298
H	3.11160352	-2.36965558	-2.14778446

Coordinates of **3d**:

C	-0.70282276	6.03879132	0.06119987
C	0.72711353	6.03649459	0.04704287
C	1.41866537	4.85830532	0.04629758
C	0.73923414	3.58995211	0.05559451
C	-0.72165149	3.59235998	0.04943741
C	-1.39768795	4.86253289	0.06147985
C	1.44633306	2.38206273	0.06892810
C	0.73342555	1.15012215	0.06518392
C	-0.72298158	1.15317626	0.05409112
C	-1.43233354	2.38657306	0.04059994
N	1.41905405	-0.00459050	0.04907022
C	0.72734714	-1.15561576	0.02936567
C	-0.72902348	-1.15098678	0.03790706
N	-1.41309402	0.00291511	0.04672679
C	1.43349427	-2.39004277	-0.02904756
C	0.71974817	-3.59326443	-0.07377457
C	-0.74114310	-3.58789998	-0.06457156
C	-1.44501423	-2.37989377	-0.00037625
C	1.39207088	-4.86449312	-0.12276134
C	0.69387797	-6.03763576	-0.17455654
C	-0.73611247	-6.03145608	-0.18390341
C	-1.42435557	-4.85255810	-0.12969130
C	-2.92797753	2.35774711	0.00512840
C	2.94211818	2.35372306	0.08821823
C	-2.94072096	-2.34397441	0.02387431
C	2.92924138	-2.36782360	-0.05897829
C	3.61661915	-2.55448567	-1.26481806
C	5.01252948	-2.53664338	-1.29328636
C	5.74504561	-2.32723230	-0.12290539
C	5.05908876	-2.13107010	1.07683587
C	3.66271733	-2.14844721	1.11146839
C	3.66312591	1.96661527	-1.04577704
C	5.05980307	1.93855640	-1.01872542
C	5.75756484	2.30599522	0.13263534
C	5.03736739	2.69265524	1.26527475
C	3.64165065	2.70650967	1.24939116
C	-3.61574314	2.66398728	-1.17547345
C	-5.01154018	2.62234707	-1.21164187
C	-5.74167401	2.26476261	-0.07673229

C	-5.05480984	1.95173972	1.09782608
C	-3.65988575	1.99747545	1.14176446
C	-3.65989763	-1.92150555	-1.09844406
C	-5.05539795	-1.86810924	-1.06404492
C	-5.75433146	-2.24372524	0.08394333
C	-5.03638909	-2.66901550	1.20430454
C	-3.64099029	-2.71032738	1.18066403
H	-1.23695887	6.98378325	0.07175306
H	1.26426264	6.97982844	0.04099435
H	2.50228452	4.86708237	0.03871795
H	-2.48123456	4.87362668	0.07478478
H	2.47563345	-4.87970682	-0.11677571
H	1.22571362	-6.98328328	-0.21121323
H	-1.27561568	-6.97215823	-0.23326667
H	-2.50801433	-4.85718013	-0.13795853
H	3.06081113	-2.71246906	-2.18284932
H	6.82825467	-2.31010476	-0.14723513
H	3.14350644	-1.98742027	2.05011791
H	3.13423689	1.67565235	-1.94700821
H	6.84102689	2.28685654	0.14966675
H	3.09560956	3.00226679	2.13851284
H	-3.06123322	2.93441040	-2.06780613
H	-6.82420000	2.22733881	-0.10843762
H	-3.13953245	1.74355540	2.05884469
H	-3.12970851	-1.62023030	-1.99544165
H	-6.83707542	-2.20331354	0.10760467
H	-3.09640262	-3.03428121	2.06092404
C	5.81323867	1.52843176	-2.25901956
C	5.78625513	3.15551489	2.49060831
C	5.82447940	-1.91747706	2.35845773
C	5.74136635	-2.79500280	-2.58873415
C	-5.80660713	-1.41688902	-2.29122862
C	-5.79055539	-3.14334406	2.42254322
C	-5.82311683	1.57061125	2.33812107
C	-5.74268427	3.01722917	-2.47116230
F	-5.14263283	0.67354325	3.09099803
F	-7.02467616	1.02331653	2.04654915
F	-6.06111445	2.64780010	3.13251089
F	-5.03947902	-3.09976712	3.54648801
F	-6.19616959	-4.43409265	2.27445586
F	-6.90529171	-2.41134136	2.64569301
F	-7.03117091	-0.92952592	-1.98987020
F	-5.13759083	-0.44486312	-2.95647444
F	-5.99246550	-2.43702603	-3.17049210
F	-6.92480242	2.37428315	-2.59571403
F	-6.01831735	4.35016236	-2.48174252

F	-5.01895362	2.76289615	-3.58597142
F	5.20668387	-1.02049525	3.16436118
F	5.93879095	-3.06911220	3.07218657
F	7.07838744	-1.46517001	2.13665223
F	6.01640570	-4.11899914	-2.74379840
F	5.01666416	-2.41903176	-3.66791754
F	6.92415746	-2.14319496	-2.64515719
F	5.18738895	0.52175641	-2.91527826
F	7.06725477	1.10867861	-1.97921723
F	5.92881506	2.55818344	-3.13925255
F	6.16076625	4.45882895	2.37398369
F	6.91873181	2.44457683	2.69109252
F	5.04067050	3.06474480	3.61528085
